# Lipid metabolism reprogramming: key mechanism of breast cancer endocrine therapy resistance

**DOI:** 10.3389/fonc.2026.1848165

**Published:** 2026-09-11

**Authors:** Zhiqi Sui, Xiaoxi Han, Shaochun Liu, Yuhan Tang, Han Zhang, Linlin Fan, Chang Su, Wenhui Zhao

**Affiliations:** Department of Medical Oncology, Harbin Medical University Cancer Hospital, Harbin Medical University, Harbin, Heilongjiang, China

**Keywords:** endocrine therapy resistance, ER+ breast cancer, fatty acid synthesis, fatty acid β-oxidation, lipid metabolic reprogramming

## Abstract

ER+ (estrogen receptor positive) breast cancer is the most common subtype of breast cancer, for which endocrine therapy is the primary treatment. However, the development of drug resistance severely limits the clinical efficacy. In recent years, lipid metabolism reprogramming, as a key metabolic feature of tumor cells adapting to microenvironmental stress and driving progression and metastasis, has gained increasing attention due to its role in promoting endocrine therapy resistance. This review focuses on the lipid metabolism reprogramming that occurs in ER+ breast cancer cells in response to endocrine therapy, systematically elaborating on the key molecular mechanism changes in fatty acid synthesis, cholesterol metabolism, lipid droplet homeostasis, and mitochondrial fatty acid β-oxidation. These metabolic remodeling processes not only provide survival advantages and energy to drug-resistant cells but also directly promote the formation and development of drug-resistant phenotypes by affecting signal transduction and epigenetic regulation pathways. By integrating the latest research progress in this field, this article aims to deeply reveal the intrinsic connection between lipid metabolism reprogramming and endocrine resistance, providing important theoretical basis and direction for overcoming the bottleneck of drug resistance and developing new combination therapy strategies targeting lipid metabolism.

## Highlights

- Reprogramming of lipid metabolism drives endocrine resistance in ER+ breast cancer.
- Key pathways involved include altered fatty acid synthesis and uptake, cholesterol metabolism, and β-oxidation.
- Targeting lipid metabolism provides promising strategies to overcome resistance to endocrine therapy.

## 1 Introduction

ER+ (estrogen receptor positive) breast cancer constitutes approximately 70% of all breast cancer cases, and its standard treatment relies on endocrine therapy that targets pathways such as estrogen signaling (1). Although initial endocrine therapy often proves effective, primary and acquired resistance ultimately lead to treatment failure in nearly 50% of patients, which poses a significant clinical challenge (1). The mechanisms of endocrine resistance are diverse. They generally encompass ESR1 mutations, activation of the PI3K/AKT/mTOR (phosphatidylinositol 3-kinase/protein kinase B/mammalian target of rapamycin) signaling pathway, alterations in cell cycle checkpoint regulation, and epigenetic modifications (2, 3)—for example, point mutations in the ligand-binding domain of the ESR1 gene, such as Y537S and D538G, frequently occur in metastatic breast cancer. These mutations result in increased transcriptional activity of ERα independent of estradiol presence, leading to resistance to traditional endocrine therapy (4, 5). In addition, the abnormal activation of the PI3K/AKT/mTOR pathway is a significant contributor to drug resistance. This pathway interacts complexly with ER signaling, and combining its inhibitors with endocrine therapy represents an important strategy to overcome resistance (6, 7). Alterations in cell cycle regulation, particularly the use of CDK4/6 (cyclin-dependent kinase 4/6) inhibitors, have significantly improved patient prognosis, but issues of primary and acquired resistance persist (8).

Evidence increasingly indicates that metabolic reprogramming serves as a key driver of tumor cell survival and proliferation under therapeutic pressure (9). Metabolic reprogramming refers to the process through which cells modify their metabolic pathways to meet energy demands, thereby promoting cell survival and growth. This process not only helps cells resist external stress but also confers new functions upon them (9). In ER+ breast cancer, endocrine therapy can induce various metabolic changes in tumor cells, notably, alterations in lipid metabolism are closely associated with the development of drug-resistant phenotypes (10). Lipids are not only the main components of cell membranes and forms of energy storage but also act as signaling molecules involved in the regulation of various pro-survival pathways (10). Studies have shown that endocrine-resistant cells, such as cells resistant to TAM (tamoxifen) and FUL (fulvestrant), exhibit significant enrichment of lipid metabolism pathways and display unique lipidomic characteristics, including elevated TAG (triglyceride) levels and increased cytoplasmic lipid droplets (10). Activation of FAO (fatty acid oxidation) has also been identified as an important mechanism underlying endocrine resistance by promoting tumor cell survival through activation of the Src signaling pathway (11). These findings collectively highlight the central role of lipid metabolic reprogramming in driving endocrine resistance. This review will focus on the core of lipid metabolic reprogramming, delve into its role as a key mechanism in endocrine therapy for ER+ breast cancer, and systematically summarize the latest progress in novel therapeutic strategies derived from this mechanism (**Table 1**).

**Table 1:** Lipid metabolic pathways, key molecules, and mechanisms of resistance associated with endocrine resistance in ER+ breast cancer.

Lipid Metabolism Pathway	Key Enzymes/Regulators	Role in Endocrine Resistance	Refs.
de novo Fatty Acid Synthesis	FASN, ACC1, ACLY, SREBP1	Promotes cell proliferation and membrane remodeling; FASN upregulation is a hallmark of resistant cells.	(10, 108)
Exogenous Fatty Acid Uptake	CD36, FABPs (FABP4/5), FATPs	Adaptive shift to environmental lipid scavenging to sustain metabolic needs under stress.	(62, 68)
Cholesterol and Oxysterol Metabolism	HMGCR, LDLR, CYP27A1 (27-HC), LXR	Accumulation of cholesterol and 27-HC acts as endogenous ligands for ER/ERRα.	(47, 85)
Lipid Storage and Mobilization	Lipid Droplets (LDs), PLINs (PLIN2/3), ATGL	LDs serve as energy reservoirs and prevent lipotoxicity; LD accumulation correlates with endocrine resistance.	(10, 104)
Mitochondrial Fatty Acid β-oxidation	CPT1, CPT2, Src activation	Provides ATP and activates the Src kinase pathway to promote pro-survival signaling.	B(179, 180)

Initially, it delineates the pivotal lipid metabolism pathways implicated in resistance, including exogenous fatty acid uptake, *de novo* lipogenesis, and cholesterol metabolism. For each pathway, it enumerates the critical enzymes and regulators, such as CD36 and FATP1 for fatty acid uptake, FASN and SREBP-1 for *de novo* lipogenesis, and HMGCR for cholesterol metabolism. Subsequently, it elucidates the mechanisms by which these pathways and molecules contribute to endocrine resistance, such as sustaining ER-α activity, initiating compensatory signaling cascades, or augmenting tumor cell energy reserves. This structured framework facilitates a deeper understanding of the mechanisms underlying lipid metabolism-mediated resistance.

## 2 Metabolic background of ER+ breast cancer endocrine therapy resistance

### 2.1 Clinical status and challenges of endocrine therapy resistance

Endocrine therapy is of paramount importance as the primary treatment for ER+ breast cancer patients. Commonly used endocrine therapy drugs include SERMs (selective estrogen receptor modulators) such as TAM, SERDs (selective estrogen receptor degraders) such as FUL, and AIs (aromatase inhibitors) such as LET (letrozole). By blocking the estrogen signaling pathway through different mechanisms, these drugs constitute the cornerstone of endocrine therapy for ER+ breast cancer (12)—for example, TAM exerts antagonistic effects in breast tissue by competitively binding to ERα (13). FUL can promote the degradation of ERα. However, its poor oral bioavailability requires intramuscular injection for administration, thereby limiting its clinical application (14). AI reduce estrogen levels in the body by inhibiting the synthesis of estrogen (15). Despite their substantial clinical benefits, the emergence of drug resistance greatly compromises the long-term efficacy of these drugs.

Endocrine therapy resistance has emerged as a significant factor influencing the prognosis of patients with ER+ breast cancer. The mechanisms of endocrine resistance are diverse, generally encompassing the following factors: ESR1 mutations, activation of the PI3K/AKT/mTOR pathway, alterations in cell cycle checkpoint regulation, and epigenetic modifications (2, 3). A retrospective study of Mexican breast cancer patients reported an incidence of endocrine resistance as high as 32.5%, and it was significantly associated with advanced clinical stage, larger tumor volume, and worse lymph node status, which constitutes an important risk factor for patient mortality (16). Acquired mutations enable ERα to maintain transcriptional activity even in the absence of estrogen receptor ligands, thereby promoting tumor growth (4, 14). Moreover, dysregulated activation of the PI3K/AKT/mTOR pathway is frequently observed in drug-resistant tumors, thereby counteracting the inhibitory effects of endocrine therapy through the promotion of cell survival and proliferation processes (17). Dysregulation of cell cycle control, particularly alterations in the cyclin D-CDK4/6-Rb axis, represents a key mechanism of endocrine resistance. This discovery has also led to the combined use of CDK4/6 inhibitors and endocrine therapy (18). Epigenetic alterations, such as DNA methylation, histone modifications, and dysregulated expression of non-coding RNAs, also reshape gene expression profiles without changing the DNA sequence, thereby promoting the emergence of drug-resistant phenotypes (2)—for example, histone methyltransferase expression is upregulated in drug-resistant cells, which sustains ERα expression by modulating histone H3K4 methylation at the ESR1 gene, thereby supporting the growth of TAM-resistant cells (19).

In addition, the occurrence of drug resistance is closely associated with intercellular interactions within the TME (tumor microenvironment). Cells in the TME, particularly tumor-associated macrophages, are critical mediators of endocrine resistance. Studies have shown that during the process of lymph node metastasis, tumor-associated macrophages can induce tumor cells to downregulate hormone receptor expression and upregulate HER2 (human epidermal growth factor receptor 2) expression, thereby promoting tumor transformation into more aggressive subtypes and contributing to resistance associated with TAM activity (20). Exosomes, key mediators of intercellular communication in the TME, play a critical role in mediating breast cancer treatment resistance, including endocrine resistance, through the delivery of bioactive molecules, including nucleic acids, proteins, and lipids (21). The metabolic state of the TME also has a profound influence on treatment response—for example, the hypoxic tumor microenvironment can induce EMT (epithelial–mesenchymal transition), a process by which epithelial cells acquire mesenchymal properties and reduce the expression of estrogen receptor ER-related markers, which leads to resistance to endocrine therapy (22). Transcriptomic evidence suggests that the EMT phenotype is closely associated with endocrine therapy resistance in ER+ breast cancer. Moreover, deep learning algorithms trained on hematoxylin-and-eosin-stained slides can accurately predict the EMT phenotype and endocrine therapy response, thereby offering a novel approach for clinical evaluation of drug resistance risk (23). Metabolic reprogramming occurs in the TME—for example, enhanced FAO has been identified as a metabolic adaptation mechanism for endocrine resistance in ER+ breast cancer. Additionally, activation of the Src signaling pathway promotes the development of drug resistance (11). These findings collectively reveal the central role of the TME and its metabolic interactions in shaping the endocrine-resistant phenotype, highlighting avenues for the development of new therapeutic strategies.

### 2.2 Prevalence of metabolic reprogramming in cancer drug resistance

Metabolic reprogramming is an adaptive process undergone by cancer cells in response to microenvironmental changes. This process is now widely recognized as one of the core hallmarks of cancer and plays a critical role in the development of drug resistance in various types of cancer (24). Metabolic adaptation is not a change in one single pathway but rather a systemic reprogramming process involving multiple metabolic pathways such as glucose, amino acids, and lipid metabolism (24). In hepatocellular carcinoma, hypoxia-inducible-factor-mediated metabolic reprogramming is a crucial molecular mechanism contributing to drug resistance (25). In breast cancer, metabolic reprogramming, such as increased rates of glucose metabolism, fatty acid synthesis, and glutamine metabolism, has been identified as a significant contributor to chemotherapy resistance (26). This reprogramming process is jointly regulated by various factors such as oncogenic signaling, inactivation of tumor suppressor genes, and non-coding RNA, which, in turn, promote cancer stemness, immune escape, and an enhanced anti-apoptotic capacity (24). A deep understanding of metabolic reprogramming, which serves as a universal adaptive strategy for cancer cells to cope with therapeutic stress, is crucial to revealing the mechanisms of drug resistance and to developing novel therapies.

Lipid metabolic reprogramming, a key aspect of cancer metabolism, has been widely shown to be crucial in tumor occurrence, development, metastasis, and treatment resistance. Changes in lipid metabolism affect not only energy supply but also intracellular signaling pathways (27). Across various cancers, dysregulation of lipid metabolism, including alterations in fatty acid synthesis, uptake, oxidation, and storage, supports the rapid growth and survival of tumor cells and directly contributes to the development of drug-resistant phenotypes (28). In breast cancer, alterations in lipid metabolism play a crucial role in tumor growth, progression, metastasis, and drug resistance. Key changes include increased uptake of fatty acids and cholesterol, enhanced biosynthesis of these lipids, as well as upregulation of FAO (29). In ovarian cancer, it does so by increasing energy production, synthesizing novel lipid metabolites, and activating relevant signaling pathways to influence tumor proliferation and migration and contribute to drug resistance development (30). Furthermore, the remodeling of lipid metabolism directly influences the diffusion and efficacy of drugs within cells by affecting cell membrane permeability, mitochondrial function, and lipid composition. This process thereby promotes the development of drug resistance (31). Therefore, targeting lipid metabolism is a promising therapeutic strategy for overcoming cancer drug resistance.

The occurrence of drug resistance is related to intercellular interactions within the TME. Tumor-associated macrophages and cancer-associated fibroblasts in the TME promote the survival and proliferation of tumor cells by secreting various factors, thereby exacerbating drug resistance. The TME is a complex ecosystem in which non-malignant cells, such as cancer-associated fibroblasts, profoundly influence tumor progression and drug resistance through metabolic reprogramming (32)—for example, cancer-associated fibroblasts can affect tumor proliferation, migration, invasion, metastasis, and drug resistance through alterations in their metabolic patterns (32). In ovarian cancer, the TME further shapes the metabolic adaptation of cancer cells, enabling them to tolerate hypoxia and nutrient deprivation. Studies using organoid models have revealed the association between metabolic reprogramming and immune evasion, such as the role of sphingolipid signaling in tumor proliferation and the role of kynurenine metabolism in the suppression of CD8+ T cells (33). Extracellular vesicles, important mediators of intercellular communication, also play a key role in metabolic reprogramming and in promoting the spread of drug-resistant phenotypes (34). These vesicles can transfer metabolic reprogramming traits from drug-resistant cells to sensitive cells, thereby amplifying drug resistance within the TME (27). Therefore, abnormal metabolic crosstalk between tumor cells and other components in the microenvironment collectively constructs multiple barriers that weaken the efficacy of chemotherapy, targeted therapy, and immunotherapy (35). Interventions targeting TME-dependent metabolic reprogramming hold promise as a new approach to overcoming resistance to anticancer drugs.

### 2.3 A paradigm shift in cellular energy utilization: from carbohydrate metabolism to lipid metabolism

In the field of breast cancer metabolism research, early studies initially concentrated on aerobic glycolysis, a phenomenon commonly observed in tumor cells known as the “Warburg effect.” However, recent evidence suggests that, especially in ER+ breast cancer under the pressure of endocrine therapy, cancer cells undergo significant metabolic reprogramming. The core feature of this reprogramming is a shift from glucose reliance toward enhanced lipid metabolism to meet their bioenergetic and biosynthetic demands (36). This metabolic shift is a key strategy for cancer cells to adapt to therapeutic pressure and maintain survival—for instance, in ER+ breast cancer, endocrine therapies such as TAM or AI alter the energy requirements of tumor cells, prompting them to seek alternative metabolic pathways (37). Studies have shown that breast cancer cells, especially those with aggressive subtypes, can support their rapid proliferation and metastasis by enhancing lipid synthesis and utilization (38). This metabolic switch is not limited to primary tumors; unique metabolic characteristics are also observed in metastatic lesions, which help cancer cells survive in harsh microenvironments and resist treatment (36).

Therefore, understanding the transition from glycometabolism to lipid metabolism is crucial for elucidating the mechanisms underlying endocrine therapy resistance. After endocrine therapy with drugs such as TAM or AI, the mitochondrial function of ER+ breast cancer cells is adaptively enhanced with upregulated OXPHOS (oxidative phosphorylation) activity (36). This change provides essential energy and supports lipid metabolism processes such as FAO. Mitochondria, as the “powerhouses” of cells, enhance their function, enabling cancer cells to more efficiently utilize substrates such as lipids for β-oxidation, thus producing large amounts of ATP (36). This metabolic remodeling contrasts sharply with the traditional Warburg effect, which primarily relies on less efficient glycolysis for energy production. Studies have confirmed that under the pressure of endocrine therapy, cancer cells reprogram hepatocyte metabolism by activating pathways such as peroxisome proliferator-activated receptor γ coactivator-1α, making cancer cells inclined toward lipid oxidation and autophagy (39). Additionally, the maintenance of mitochondrial membrane potential and the normal operation of electron transport chain activity are crucial for FAO. Evidence shows that certain environmental pollutants (such as diphenyl phosphate) impair mitochondrial function, leading to decreased membrane potential, inhibition of electron transport chain activity, and a significant reduction in ATP production capacity, which conversely underscores the importance of intact mitochondrial function for lipid energy metabolism (40). Therefore, the enhancement of mitochondrial function and the upregulation of OXPHOS activity induced by endocrine therapy are core mechanisms driving lipid metabolic reprogramming and supporting cancer cell survival.

This metabolic shift from glucose dependence to enhanced utilization of lipid substrates enables ER+ breast cancer cells to maintain survival under nutrient-limited or therapeutic pressure (36). When glucose supply is insufficient or glycolysis pathways are inhibited, cancer cells can flexibly switch to utilizing circulating lipids or mobilizing endogenous lipid reserves—for example, studies have indicated that under glucose-deficient conditions, certain cancer cell lines enhance the uptake of palmitic acid and direct it toward lipid synthesis pathways, such as synthesizing TAG, to store energy and build biological membranes (41). This metabolic flexibility endows cancer cells with strong adaptive capabilities. Specifically, lipids not only serve as efficient energy sources for ATP production through β-oxidation but their metabolic intermediates can also act as signaling molecules or biosynthetic precursors, supporting cell proliferation (38). In the TME, the interaction between cancer cells and adipocytes further exacerbates this metabolic dependence. In obesity-related breast cancer, accumulated adipose tissue releases fatty acids and adipokines, providing abundant lipid substrates for tumor cells and thereby promoting tumor progression (42). Therefore, enhancing the utilization of lipid substrates is an important mechanism for cancer cells to overcome metabolic challenges caused by endocrine therapy or microenvironmental pressure, enabling sustained growth and the development of treatment resistance.

## 3 Upregulation of the *de novo* fatty acid synthesis pathway and its role in drug resistance mechanisms

### 3.1 Regulation of the expression levels and enzymatic activities of key enzymes

FASN is highly expressed in most ER+ breast cancers, and its expression level is positively correlated with endocrine therapy resistance and poor prognosis. Studies have shown that in Luminal B breast cancer cell models simulating TAM/FUL resistance, HRG (autocrine heregulin) upregulates FASN expression by activating the HER2/PI3K/AKT and MAPK-ERK1/2 (mitogen-activated protein kinase–extracellular signal-regulated kinase 1/2) signaling pathways (43). Inhibiting FASN activity can restore the anti-tumor activity of TAM and FUL against resistant tumors (43). High FASN expression not only promotes tumor cell proliferation and migration but also influences tumor invasion and metastasis by regulating the tumor immune microenvironment and contributing to EMT (44). In various tumor types, an aberrant FASN expression has been confirmed to be highly associated with tumor migration and invasion (44). Therefore, FASN is a key biomarker for endocrine resistance in ER+ breast cancer and a potential therapeutic target. Inhibitors targeting FASN may become a novel strategy to reverse endocrine therapy resistance and improve the prognosis of Luminal B breast cancer patients.

ACC (acetyl-CoA carboxylase) is the rate-limiting enzyme in fatty acid synthesis. Its activity is regulated by signaling pathways such as AMPK (AMP-activated protein kinase)/mTOR and is often activated in endocrine-resistant cells. AMPK, acting as a cellular energy sensor, phosphorylates and inhibits ACC activity, thereby reducing fatty acid synthesis (45). Conversely, activation of the mTOR signaling pathway tends to promote lipid synthesis. Research indicates an interaction between the transcription factor SREBP1 (sterol regulatory element-binding proteins) and the α1 subunit of AMPK (AMPK-α1), which jointly regulates lipid biosynthesis. In the filamentous fungus *Mucor circinelloides*, simultaneous overexpression of sre1 (the SREBP1 homolog) and knockout of AMPK-α1 significantly upregulates the expression and enzymatic activity of key lipogenic genes, including ACC, thereby substantially increasing lipid yield (45). Additionally, lipidomics studies have pointed out that ACC is one of the core enzymes in breast cancer metabolic reprogramming, and changes in its activity are closely related to various tumor types (46). Therefore, a deep understanding of the molecular mechanisms by which ACC is regulated by pathways such as AMPK/mTOR in endocrine-resistant cells is of great significance for developing novel therapies targeting the lipid synthesis pathway to overcome treatment resistance.

ACLY (ATP-citrate lyase) is a key enzyme bridging cellular glucose metabolism and lipid anabolism. It catalyzes the cleavage of mitochondrially derived citrate into cytosolic acetyl-CoA and oxaloacetate, thereby providing the essential acetyl group that serves as a precursor for the *de novo* synthesis of fatty acids and cholesterol. In breast cancer, particularly in endocrine therapy-resistant models, the expression and the activity of ACLY are often significantly upregulated—for example, in TAM-resistant MCF-7 cells, OHT promotes ACLY expression and activity by modulating signaling pathways, thereby accelerating lipid synthesis. OHT (4-hydroxytamoxifen) treatment activates the metabolic pathway from citrate to lipids, during which the expression of ACLY and its downstream fatty acid biosynthetic enzymes is significantly elevated (47). This metabolic reprogramming is crucial for inducing cancer cell death. The combined use of an ACLY inhibitor and TAM can decrease cell viability and lower mitochondrial membrane potential, indicating that the ACLY-mediated lipid synthesis pathway plays a key role in drug-induced cell death (47). Furthermore, within the regulatory network of lipid biosynthesis, ACLY activity is also influenced by upstream signaling molecules. Research shows that in *Mucor circinelloides*, overexpression of the transcriptional regulator SREBP1 (sre1) can upregulate the mRNA levels and enzymatic activity of multiple key lipogenic genes, including acl, acc1, and fas1, thereby providing more precursors for lipid biosynthesis (45). This suggests that in the context of endocrine resistance in breast cancer, similar transcriptional regulatory mechanisms may drive high ACLY expression.

### 3.2 Functional consequences of synthetic pathway activation

Enhanced fatty acid synthesis provides abundant precursors for membrane phospholipids to rapidly proliferating drug-resistant cells, supporting their high proliferation rate (48). This metabolic reprogramming is a hallmark of cancer, involving enhancement of multiple metabolic pathways, including fatty acid synthesis (49). Specifically, drug-resistant cells upregulate *de novo* lipid synthesis pathways to supply sufficient phospholipid precursors for the rapid renewal and expansion of cell membranes (50). Studies have shown that in breast cancer, the activity of key enzymes such as FASN is significantly enhanced, which directly supports the high proliferation rate of drug-resistant cells (51)—for example, in HER2+ breast cancer brain metastasis models, fatty acid synthesis has been demonstrated to be essential for tumor growth in the brain microenvironment. This highlights the central role of lipid synthesis in supporting tumor cell proliferation within specific niches (52). Furthermore, the reprogramming of lipid metabolism is not limited to fatty acid synthesis but also involves complex regulatory networks. Transcription factors such as SREBP-1 are aberrantly activated in obesity-induced breast cancer, driving lipid synthesis and thereby promoting tumor growth and metastasis (53). This enhanced lipid synthesis capability provides drug-resistant cells with the necessary structural components for constructing new cell membranes, serving as a crucial material foundation for maintaining their malignant phenotype and resisting therapeutic pressure (54).

Newly synthesized fatty acids are converted into lipid signaling molecules, such as lysophosphatidic acid and phosphatidic acid, which can transactivate the ER or downstream MAPK/PI3K pathways (48). In ER+ breast cancer, when the traditional estrogen signaling pathway is blocked by endocrine drugs, cancer cells utilize these lipid signaling molecules to activate alternative pathways, thereby bypassing therapeutic inhibition. Specific mechanisms include the ability of these lipid molecules to transactivate the ER or directly stimulate its downstream key signaling pathways, such as the MAPK and PI3K pathways (55). Studies have shown that in TAM-resistant cells, tafazzin deficiency leads to the upregulation of lysophosphatidylcholine levels, and its further metabolite, lysophosphatidic acid, as a bioactive molecule, can support cell survival and potentially promote drug resistance by affecting the expression and localization of ERα (56). Furthermore, there is a complex interplay between lipid metabolism and oncogenic signaling pathways—for example, the RAS gene can regulate lipid metabolism through pathways like AKT-mTORC1, and conversely, lipid metabolism can modify and activate RAS proteins and their downstream signaling pathways (57).

Activation of the fatty acid synthesis pathway not only provides membrane precursors and signaling molecules but also, through an important post-translational modification—protein palmitoylation—directly influences the function of ERα and its coregulators, thereby regulating gene expression at the transcriptional level. Protein palmitoylation refers to the covalent attachment of a palmitoyl group to cysteine residues, a modification that can significantly alter membrane localization, stability, protein–protein interactions, and the activity of target proteins (48). In breast cancer, ERα and its coregulators are potential targets of palmitoylation. This lipid modification can affect the subcellular localization of ERα, such as promoting its recruitment to the cell membrane or specific membrane microdomains, thereby participating in non-genomic signaling (58). More importantly, palmitoylation can regulate the transcriptional activity of ERα. By modifying ERα itself or its coactivators/corepressors, palmitoylation can alter the binding efficiency to estrogen response elements or the ability to recruit transcriptional complexes, directly regulating the transcription of a series of genes that drive cell proliferation and survival (53). Research indicates that reprogramming of lipid metabolism, particularly changes in fatty acid synthesis and cholesterol metabolism, can affect the composition and function of cell membrane lipid rafts, which are platforms for the aggregation of many signaling proteins, including growth factor receptors (58)—for example, PGRMC1 influences the membrane localization and activation of receptors such as EGFR (epidermal growth factor receptor) and ERα by altering lipid metabolism and lipid raft formation, driving breast cancer progression (58). Additionally, palmitic acid, as a substrate for palmitoylation, directly increases the likelihood of protein palmitoylation when its intracellular levels rise. In drug-resistant cells, enhanced fatty acid synthesis ensures an adequate supply of palmitic acid, potentially leading to sustained modification and activation of the ERα signaling network. This maintenance supports its pro-transcriptional function even under conditions of estrogen deprivation or in the presence of receptor antagonists (59).

## 4 Adaptive changes in the uptake and metabolism of exogenous fatty acids

### 4.1 Role of FATPs

FATPs (fatty acid transport proteins) play a critical role in tumor metabolic reprogramming. CD36, as an important long-chain FATP, is upregulated in endocrine-resistant cell lines and patient relapse samples, promoting lipid uptake from the microenvironment. Studies show that in the TME, especially in adipose tissue-rich environments, breast cancer cells increase exogenous lipid uptake through the upregulation of CD36 expression to meet the energy and biosynthetic demands for rapid proliferation and survival (60). This enhanced uptake capacity enables cancer cells to acquire large amounts of long-chain fatty acids from the surrounding environment, thereby supporting their metabolic reprogramming and maintenance of drug-resistant phenotypes. In analyses of clinical samples, CD36 expression is significantly elevated in tumor tissues from patients with endocrine therapy resistance or relapse, suggesting that CD36 may serve as a potential biomarker for predicting endocrine therapy response and disease progression (61). Mechanistically, CD36-mediated lipid uptake not only provides energy substrates for cancer cells but may also influence cell fate by affecting downstream signaling pathways, such as regulating cell death processes like ferroptosis (60). Furthermore, CD36 function is finely regulated by post-translational modifications—for example, palmitoylation is crucial for its mediation of monounsaturated fatty acid uptake, maintenance of lipid homeostasis, and prevention of saturated fatty acid-induced lipotoxicity. This process is particularly important during tumor cell detachment from the matrix and metastasis (62). Therefore, the upregulation of CD36 expression becomes one of the key mechanisms by which breast cancer cells adapt to metabolic stress and gain survival advantages, ultimately leading to therapy resistance.

In addition to membrane transporters like CD36, the intracellular FABPs (fatty acid-binding proteins) family also plays an indispensable role in lipid metabolic reprogramming. FABPs are a class of intracellular lipid chaperone proteins with a molecular weight of approximately 14 to 15 kDa. They precisely regulate metabolic pathways, signal transduction, and gene expression by binding and transporting fatty acids and other lipid molecules (63). In cancer, the expression profile of FABPs is significantly altered, enhancing intracellular fatty acid transport efficiency and promoting the delivery of fatty acids to metabolic sites such as mitochondria and the endoplasmic reticulum, thereby supporting malignant tumor progression (64). In breast cancer, overexpression of FABP3 has been shown to be oncogenic, and its knockdown can inhibit cancer cell proliferation, migration, and invasion (65). FABP isoforms exhibit tissue specificity, and their functions are diverse. FABP5 in pancreatic cancer enhances intracellular bile acid transport, activating the NR1H2/3 signaling pathway to promote lipid metabolic reprogramming and tumor metastasis (66). In colorectal cancer, loss of FABP1 promotes a shift in metabolism toward oxidative pathways, inhibiting fatty acid and cholesterol synthesis (67). These alterations in expression profiles enable cancer cells to utilize endogenous and exogenous lipids more efficiently to meet their needs for biomembrane synthesis, energy production, and signaling molecule generation. Additionally, FABPs are involved in regulating the immune microenvironment—for example, FABP4+ tumor-associated macrophages can inhibit T cell activity by secreting IL-6, while FABP6 can downregulate MHC-I molecule expression to reduce CD8+ T cell infiltration, thereby creating an immunosuppressive environment and promoting therapy resistance (63). Therefore, changes in FABP expression profiles are central to breast cancer cells achieving lipid metabolic reprogramming, maintaining survival, and acquiring drug resistance.

Inhibiting CD36 can partially restore sensitivity to endocrine therapy, which indicates that exogenous lipid uptake is necessary for maintaining the drug-resistant phenotype. Experimental evidence shows that inhibiting CD36 expression or function can partially restore cancer cell sensitivity to endocrine therapy, thereby directly demonstrating that exogenous lipid uptake is a prerequisite for maintaining the drug-resistant phenotype (68). In preclinical models of breast and ovarian cancer, inhibiting FATPs, including CD36, can resensitize cancer cells to oncolytic virus therapy by regulating lipid flow in the TME (68). Specifically, when FATP inhibitors or genetic knockdown methods are used to inhibit CD36, cancer cells’ ability to take up lipids from the microenvironment decreases, leading to depletion of intracellular lipid pools, impaired energy supply and biosynthesis, and ultimately weakening their proliferation, survival, and metastatic capabilities (69). In TNBC (triple-negative breast cancer) cells, simultaneous inhibition of CD36 and Nogo-B expression synergistically suppresses cell proliferation and migration. The mechanism involves reducing the expression of FABP4 and FATP4, thereby inhibiting fatty acid uptake and transport and activating the P53-P21-Rb signaling pathway (69). Furthermore, loss of CD36 in breast cancer mouse models can mitigate the metastasis-promoting effects of a high-fat diet, and only palmitoylation-competent CD36 can rescue this metastasis-promoting phenotype, further highlighting the therapeutic potential of targeting CD36 and its modifications (62). These findings collectively indicate that targeting CD36 can not only directly cut off the “lipid fuel” supply to cancer cells but may also overcome drug resistance by affecting downstream cell death pathways (such as ferroptosis) and signal transduction networks (60).

### 4.2 Lipid supply in the TME

In ER+ breast cancer, stromal cells within the TME, particularly cancer-associated fibroblasts and adipocytes, act as key external providers of lipid fuel to cancer cells (70) (71). Specifically, adipocytes release FFAs through lipolysis, which cancer cells subsequently uptake—for instance, in lymph node metastases of head and neck squamous cell carcinoma, the hypoxic TME promotes adipocyte apoptosis and necrosis, accelerating TAG release. Tumor cells utilize these released TAG to promote lipid droplet accumulation and induce chemotherapy resistance (72). Furthermore, adipocytes can secrete exosome-encapsulated lipids, providing “ready-made” lipids for cancer cells. The adipocyte secretome is a significant reservoir of regulatory factors, influencing tumor cell behavior by secreting numerous cytokines, adipokines, lipid metabolites, and exosome-encapsulated substances (73). Research indicates that blocking cancer cell uptake of adipocyte-derived lipids or interfering with related lipid metabolic pathways may become effective strategies against cancers growing in lipid-rich microenvironments (71). Therefore, targeting lipid transfer between adipocytes and cancer cells in the TME provides new intervention ideas for overcoming endocrine therapy resistance.

Tumor-associated macrophages are one of the most abundant stromal cell types in the TME, participating in lipid supply through unique metabolic reprogramming to support cancer cell growth and immune evasion (74). In lipid-enriched TMEs, tumor-associated macrophages can phagocytose excess extracellular lipids and modified low-density lipoprotein particles, transforming into dysfunctional lipid-laden cells (75). The accumulation of lipid-laden tumor-associated macrophages is a common feature in atherosclerosis, obese tissues, and the TME. When tumor-associated macrophages clear non-apoptotic cellular debris, they release lipid components, including cholesterol and fatty acids, which can subsequently be utilized by neighboring cancer cells—for example, in colorectal cancer, tumor-secreted GRP78 (glucose-regulated protein 78) can enter tumor-associated macrophages and promote their lipolysis, generating fatty acids such as arachidonic acid and docosahexaenoic acid. These fatty acids not only provide energy for the tumor-associated macrophages themselves but also mediate TAM polarization toward the M2 phenotype via PPARγ (peroxisome proliferator-activated receptor γ) activation, thereby maintaining the tumor’s immunosuppressive microenvironment (76). Additionally, in CD5-positive non-MYC/BCL2 double-expressing diffuse large B-cell lymphoma, enhanced lipid metabolism, including fatty acid uptake and oxidation, provides energy for the polarization and activation of M2 tumor-associated macrophages (77). This metabolic reprogramming allows tumor-associated macrophages not only to clear cellular debris but also inadvertently provide alternative lipid resources for cancer cells.

The metabolic symbiotic relationship between cancer cells and stromal cells in the TME is a key strategy for cancer cells to maintain survival and proliferation when facing limitations in endogenous lipid synthesis—for example, when the endogenous lipid synthesis pathways of cancer cells are targeted and inhibited through endocrine therapy or specific metabolic inhibitors, they can instead “plunder” lipid resources provided by other cells in the microenvironment (78). This plundering behavior relies on complex intercellular interactions and metabolic reprogramming—for instance, in pancreatic ductal adenocarcinoma, when tumor growth exceeds local nutrient supply, cancer cells heavily depend on the sterol regulatory element-binding protein pathway to activate genes related to lipid synthesis and uptake, and inhibiting this pathway prevents cancer cell growth under low-serum conditions due to insufficient lipid supply (79). This, in turn, highlights the importance of external lipid supply when endogenous synthesis is blocked. In breast cancer, bidirectional signaling exists between cancer cells and adipocytes, forming a metabolic symbiotic relationship (71). Stimulated by the tumor, adipocytes undergo lipolysis, and the released fatty acids are taken up by cancer cells via specific receptors such as CD36 and FATP1 for membrane synthesis, energy production, or synthesis of lipid-derived signaling molecules (71). Similarly, tumor-associated macrophages, through lipid metabolic reprogramming, release lipids while clearing cellular debris, which are also utilized by cancer cells (75). This metabolic reciprocity enables cancer cells to adapt to harsh microenvironments and evade therapeutic pressure. The heterogeneity and metabolic plasticity of the TME further strengthen this symbiotic relationship, as different cell types can adjust their metabolic output based on nutrient availability (80). Therefore, even if the autonomous lipid synthesis capacity of cancer cells is successfully inhibited, they may still survive through “ready-made” supplies in the microenvironment, constituting another important mechanism of endocrine therapy resistance. Targeting various aspects of this metabolic symbiotic relationship, such as interfering with the function of the lipid transporter CD36 or disrupting intercellular metabolic connections, may become effective new strategies for overcoming drug resistance.

## 5 Abnormal cholesterol metabolism and drug resistance

### 5.1 Imbalance in cholesterol synthesis and uptake

The upregulation of HMGCR (3-hydroxy-3-methylglutaryl-CoA reductase) activity directly promotes *de novo* cholesterol synthesis within cells. This supplies tumor cells with necessary structural components for membrane construction and maintains membrane fluidity and stability, both vital for cell division and signal transduction (81). Multiple signaling pathways cooperate in breast cancer cells to promote HMGCR expression and activity—for instance, NUCB2 (nucleobindin 2)/nesfatin-1 upregulates cholesterol synthesis genes, including HMGCR, by activating the mTORC1–SREBP2 signaling axis, thereby driving breast cancer metastasis (82). Furthermore, DHCR24 (24-dehydrocholesterol reductase) has been identified as a key driver of breast cancer progression; its high expression is closely associated with poor patient prognosis and may function by influencing the cholesterol synthesis pathway (83). In ER+ breast cancer cells, KDM5B (lysine demethylase 5B) collaborates with the CRL4B (Cullin4B–RING ubiquitin ligase) complex to epigenetically suppress the expression of INSIG1/2 (insulin-induced gene 1/2), negative regulators of cholesterol synthesis. This relieves the inhibition on the SREBP pathway, indirectly promoting the transcription of cholesterol synthesis genes, including HMGCR, and driving tumor growth (84). These findings collectively reveal that enhanced HMGCR activity is a critical component for ER+ breast cancer cells to achieve cholesterol metabolic reprogramming and meet their biosynthetic demands.

In addition to boosting endogenous synthesis, ER+ breast cancer cells also meet their substantial cholesterol needs by increasing the uptake of exogenous cholesterol. LDLR (low-density lipoprotein receptor), the primary receptor mediating cellular uptake of esterified cholesterol from circulation via LDL, is frequently upregulated in breast cancer, thereby serving as a key pathway for tumor cells to acquire cholesterol (85). Studies have found that knockdown of IGFBP6 (insulin-like growth factor-binding protein 6) leads to a significant decrease in the expression of LDLR and its adaptor protein LDLRAP1 in MDA-MB-231 breast cancer cells, accompanied by upregulation of PCSK9 (proprotein convertase subtilisin/kexin type 9), which promotes LDLR degradation, collectively resulting in reduced cellular capacity for exogenous cholesterol uptake (86). This phenomenon suggests that LDLR expression is finely regulated in specific contexts, and its upregulation is an important strategy for tumor cells to cope with cholesterol demands. Clinical research supports this view. A window-of-opportunity trial in breast cancer patients found that using statins (e.g., atorvastatin) to inhibit cholesterol synthesis led to a significant increase in LDLR expression in tumor tissue. This likely represents a compensatory cellular response to maintain intratumoral cholesterol levels following inhibition of endogenous synthesis (87). Additionally, the FGFR signaling pathway has been found to regulate cholesterol metabolism in breast cancer cells, although its specific impact on LDLR requires further investigation (88). Therefore, increased LDLR expression enables ER+ breast cancer cells to efficiently scavenge cholesterol from the TME or circulation, working synergistically with enhanced endogenous synthesis pathways to maintain a cholesterol homeostasis imbalance favorable for tumor growth.

Cholesterol is not uniformly distributed in the cell membrane but forms microdomains called “lipid rafts” together with sphingomyelin and others. These lipid rafts serve as signal transduction platforms, capable of enriching and activating various growth factor receptors and signaling proteins (81). In ER+ breast cancer, excess cholesterol accumulated through synthesis and uptake pathways is extensively integrated into the cell membrane, altering the composition and properties of lipid rafts. This alteration holds significant pathophysiological importance because it can enrich and activate tyrosine kinase receptors such as HER2 and IGF (insulin-like growth factor)-1 receptor-1R (89). Activation of these receptors can initiate downstream potent pro-proliferation and survival signaling pathways like MAPK and PI3K/AKT. Research confirms that cholesterol can act as an endogenous ligand for ERRα, directly activating it and subsequently promoting breast cancer cell proliferation, migration, and metabolic reprogramming (85, 90). ERRα activation is associated with the upregulation of various pro-cancer metabolic genes. Furthermore, oxidized cholesterol metabolites, such as 27-HC (27-hydroxycholesterol), have been shown to promote breast cancer stem cell self-renewal, EMT, and tumorigenesis through pathways like LXR activation (91). Therefore, cholesterol accumulation not only provides structural material but also abnormally activates multiple alternative growth signaling pathways by remodeling the cell membrane lipid raft microenvironment. This provides a solid molecular basis for ER+ breast cancer cells to bypass estrogen-dependent growth restrictions and develop endocrine therapy resistance (92).

### 5.2 Oxysterols and signal transduction

Cholesterol is not only a key structural component of cell membranes but also a precursor to various bioactive molecules. Cholesterol is converted into a series of oxysterols through enzymatic oxidation reactions, among which 27-HC is one of the important side-chain oxysterols (93). They are not only intermediate products in the process of cholesterol metabolism into bile acids but also important signaling molecules (93). As a member of the nuclear receptor superfamily, LXR (liver X receptor) functions as a key cholesterol sensor and is central to maintaining systemic cholesterol homeostasis (94). Studies have shown that various oxysterols, including 27-HC, are endogenous ligands of LXR, which bind to LXR and activate its transcriptional activity (94). This activation is crucial for regulating the expression of genes related to cholesterol absorption, transport, and efflux—for example, LXR activation can upregulate the expression of ABCA1 (ATP-binding cassette transporter A1), thereby promoting reverse cholesterol transport (95). Therefore, the metabolism of cholesterol into oxysterols, particularly 27-HC, constitutes a signaling pathway that closely links intracellular cholesterol levels with the regulation of gene expression, serving as one of the core mechanisms for maintaining cellular lipid homeostasis (96).

In ER+ breast cancer, 27-HC plays a key role in connecting metabolic reprogramming with therapeutic drug resistance. 27-HC is a ligand for LXR and has also been confirmed to act as a partial agonist of ER (97). This dual activity enables 27-HC to exert unique pro-cancer effects in the TME. Research indicates that 27-HC can bind to and activate ER, driving the proliferation of ER-positive breast cancer cells (98). More importantly, even in the presence of SERMs such as TAM, 27-HC can still partially activate the ER signaling pathway, thereby diminishing the efficacy of endocrine therapy and promoting the development of drug resistance. This mechanism directly links abnormal cholesterol metabolism within tumor cells or the microenvironment to the failure of endocrine therapy (97). Additionally, the increased content of oxysterols such as 27-HC in extracellular vesicles derived from breast cancer cells, regulated by Tspan6, can reshape the tumor immune microenvironment by activating the LXR signaling axis, which may indirectly affect the tumor’s response to treatment (99). Therefore, as a functional bridge between cholesterol metabolism and the ER signaling pathway, 27-HC is a key mediator leading to endocrine therapy resistance in ER+ breast cancer, thus providing a novel target for therapeutic intervention to overcome drug resistance (98).

The activation of LXR is an important feedback regulatory mechanism for cells to manage cholesterol overload. By regulating the expression of multiple target genes, it forms a precise cholesterol homeostasis regulatory network. When intracellular cholesterol levels rise, cholesterol oxidation products, such as 27-HC, act as ligands to activate LXR (94). Activated LXR then induces the expression of cholesterol efflux-related genes, such as ABCA1 and ABCG1, promoting the transport of cholesterol from cells to apolipoproteins, thereby reducing intracellular cholesterol load (95). Simultaneously, LXR activation also inhibits *de novo* cholesterol synthesis, notably by inducing the expression of INSIG to inhibit the processing and activation of SREBP, a key transcription factor controlling the transcription of cholesterol synthesis genes (98). This multi-layered feedback regulation ensures the stability of cellular cholesterol levels. However, in pathological states such as breast cancer, this network may be disrupted or exploited—for instance, in fetal–placental endothelial cells, LXR agonists can antagonize the inflammatory response induced by oxysterols, and their protective effects partially depend on ABCA1 (95). In breast cancer, integrated multi-omics analysis has revealed that the ESR1–CH25H (cholesterol 25-hydroxylase)–INSIG1–ABCA9 axis is one of the most prominent axes in the oxysterol-related interaction network, where both INSIG1 and ABCA9 are key genes regulating cholesterol homeostasis, highlighting the central role of LXR and its downstream targets in the lipid metabolic reprogramming of breast cancer (98).

## 6 Lipid storage and mobilization: the dynamic role of lipid droplets

### 6.1 Formation and function of lipid droplets

Endocrine therapy induces a substantial accumulation of lipid droplets in ER+ breast cancer cells, serving as reservoirs for neutral lipids, mitigating lipotoxicity, and maintaining intracellular lipid homeostasis (10). Studies have shown that acquired resistant cells, such as those resistant to TAM, FUL, or long-term estrogen deprivation, exhibit a significant enrichment of lipid metabolism pathways. These cells display unique lipidomic profiles characterized by elevated TAG levels and a marked increase in cytoplasmic lipid droplets (10). This accumulation of lipid droplets is not a passive process but rather an active adaptive mechanism by which cells respond to therapeutic stress. Lipid droplets serve as central storage organelles for neutral lipids (primarily TAG and cholesterol esters), formed from the endoplasmic reticulum membrane (100). Within the endoplasmic reticulum membrane, the synthesis and the accumulation of TAG lead to the deformation of the membrane bilayer, ultimately resulting in the budding of mature lipid droplets into the cytoplasm (101). In resistant cells, this process is upregulated to cope with excess lipids generated due to enhanced FASN activity (10). By encapsulating excess FFAs and TAG within lipid droplets, cells can effectively mitigate lipotoxicity resulting from lipid overload, thereby maintaining intracellular lipid homeostasis and providing a favorable metabolic environment for tumor cell survival and proliferation (102).

The increased expression of lipid-droplet-associated proteins, such as PLIN2 and PLIN3 (perilipin 2/3), stabilizes lipid droplet structure and regulates lipid exchange. Lipid droplets are not simple lipid spheres: their surface is coated with a monolayer of phospholipids and embedded with numerous functionally diverse proteins, collectively forming the lipid droplet proteome, which precisely regulates the biogenesis, growth, stability, and catabolism of lipid droplets (103). Among lipid-droplet-associated proteins, the perilipin family proteins, particularly PLIN2 and PLIN3, play crucial roles in lipid droplet function. Studies indicate that, in ER+ breast cancer models resistant to endocrine therapy, lipid droplet accumulation is accompanied by alterations in specific protein expression profiles (10). PLIN2, as a major structural protein on the lipid droplet surface, has expression levels that closely correlate with TAG content in hepatocytes and is critical for regulating liver lipid droplet size and neutral lipid storage during fasting (104). Similarly, PLIN3 has also been shown to participate in stabilizing lipid droplet structure. In studies of prostate cancer, ACSS3 (acyl-CoA synthetase short-chain family member 3) was found to reduce lipid droplet deposition by downregulating the lipid-droplet-coating protein PLIN3, thereby inhibiting tumor progression (105). These proteins not only act as physical barriers to prevent premature hydrolysis of neutral lipids within lipid droplets by cytoplasmic lipases but also serve as scaffold proteins to recruit other regulatory factors, such as comparative gene identification-58 and adipose TAG lipase, to regulate the timing and rate of lipid mobilization (102). Therefore, the upregulation of proteins like PLIN2 and PLIN3 is a key molecular mechanism by which endocrine-resistant cells stabilize their abundant lipid droplet stores and finely balance lipid storage and utilization.

Lipid droplets can be likened to active organelles involved in signal transduction and protein degradation. Recent research has fundamentally transformed the traditional understanding of lipid droplet function, revealing their nature as highly dynamic and multifunctional organelles (106). In the context of ER+ breast cancer endocrine resistance, the active functions of lipid droplets are particularly prominent. First, lipid droplets are important metabolic signaling hubs. They form membrane contact sites with various organelles, such as interactions with mitochondria. In models of metabolic-dysfunction-associated fatty liver disease, distinct contact patterns between lipid droplets and mitochondria have been observed, participating in lipid consumption or lipid droplet expansion, respectively (107). In breast cancer cells, lipid droplet accumulation and peroxisome enrichment jointly control redox homeostasis, conferring metabolic adaptability to resistant cells (108). Second, lipid droplets are involved in cellular stress responses. They can sequester toxic lipids, reactive oxygen species, and misfolded proteins, providing cytoprotective effects (109)—for example, in the nervous system, lipid droplet accumulation is considered a protective response to oxidative stress (100). Finally, lipid droplets also play a significant role in protein degradation. In yeast, lipid droplets connect with vacuoles (analogous to lysosomes) via vCLIP contact sites, a process mediated by proteins such as Ldo16/45, which is critical for lipid droplet breakdown via microautophagy under glucose starvation conditions (110). Furthermore, evidence suggests that lipid droplets may be linked to cellular protein quality control pathways (103). In the context of cancer, this multifunctionality of lipid droplets is hijacked by tumor cells—for instance, lipid droplet accumulation regulates FAO through lipid droplet–mitochondria coupling, providing energy for breast cancer metastasis (111). Therefore, the abnormal accumulation of lipid droplets in ER+ breast cancer cells is far more than a symbol of lipid storage; it is an active functional platform driving resistance and disease progression, involving multiple layers such as metabolic reprogramming, stress resistance, and integration of cellular signaling.

### 6.2 Role of lipid droplets in stress adaptation

Under therapy-induced energy or oxidative stress, TAG stored in the lipid droplet core can be hydrolyzed by lipases like ATGL (adipose TAG lipase), releasing FFAs (free fatty acid) (112). These fatty acids subsequently enter mitochondria for β-oxidation, providing ATP to sustain basic survival and proliferation needs (113)—for example, under energy stress induced by glucose deprivation, PFKL (phosphofructokinase, liver type) is phosphorylated and translocates to lipid droplets. By phosphorylating the lipid droplet coat protein PLIN2, it promotes the binding of PLIN2 to CPT1A (carnitine palmitoyltransferase 1A), thereby physically tethering lipid droplets to mitochondria. This process recruits ATGL to this region, efficiently mobilizing lipids for β-oxidation to support tumor cell proliferation (113). Similarly, in non-small cell lung cancer, glutamine deficiency activates AMPK, which, in turn, phosphorylates CHKα2 (checkpoint kinase α2), promoting its binding to lipid droplets and recruiting ATGL and autophagosomes to drive lipid droplet lipolysis, providing fuel for cancer cell survival under nutrient stress (114). Additionally, released fatty acids can also be used for the synthesis of membrane phospholipids to repair or generate new membrane structures, which is essential for maintaining cellular integrity under therapeutic pressure. Studies have shown that in cancer cells under acute amino acid starvation, autophagy provides lipid precursors for lipid droplet biogenesis, and the DGAT1 (diacylglycerol acyltransferase 1)-mediated lipid droplet formation, together with ATGL-mediated lipolysis, constitutes a dynamic cycle that enables cells to flexibly respond to fluctuations in metabolic demands (111). Through their ability to store and mobilize lipids, lipid droplets serve as a central hub for cancer cells to maintain energy homeostasis and membrane stability under therapy-induced metabolic stress.

Lipid droplets protect cells from ferroptosis by sequestering PUFAs (polyunsaturated fatty acids), reducing their involvement in lipid peroxidation. Ferroptosis is an iron-dependent form of cell death driven by lipid peroxidation, and its core mechanism involves sequestering PUFAs, which are prone to lipid peroxidation, within the neutral TAG core of lipid droplets, thereby reducing their exposure to ROS (reactive oxygen species) (115). When cells face oxidative stress, the stabilization of the transcription factor NRF2 can upregulate the expression of G0S2, which inhibits ATGL activity, reduces the release of fatty acids from lipid droplets, and subsequently locks fatty acids inside the droplets, preventing lipotoxicity and inhibiting ferroptosis (116). This protective mechanism is particularly prominent in the TME—for example, in osteosarcoma cells, extracellular acidosis induces massive accumulation of lipid droplets, and the formation of these droplets is negatively correlated with ROS levels and lipid peroxidation, which is crucial for tumor cells exposed to acid to clear oxidative stress and survive (117). Similarly, in the acidic microenvironment of glioblastoma and central nervous system metastases, tumor cells remodel their glycocalyx to limit the uptake of lipid particles and rely on lipid droplet formation to buffer toxic lipids, thereby inhibiting ferroptosis (118). In breast cancer, the accumulation of lipid droplets has also been shown to be a key strategy for cancer cell survival under chemotherapy-induced toxic stress; cells that survive chemotherapy exhibit elevated lipid droplet levels, and these droplets can sequester damaged and excess lipids generated by oxidative stress, promoting cell survival (119). Furthermore, lipid droplets can regulate ferroptosis sensitivity by influencing enzymes involved in lipid metabolism—for instance, the endoplasmic reticulum stress sensor protein IRE1α degrades DGAT2 mRNA through its RIDD (regulated IRE1-dependent decay) activity, thereby inhibiting TAG synthesis and lipid droplet formation; inhibiting IRE1α leads to DGAT2-dependent lipid droplet accumulation and alters cellular sensitivity to nutrient stress (120). These studies collectively indicate that lipid droplets, as a “buffer pool” for lipid peroxidation, constitute an important line of defense for cancer cells against ferroptosis through both physical sequestration and metabolic regulation.

Lipid droplets are not static storage depots but highly dynamic organelles. Their biogenesis, expansion, contraction, and degradation form a finely regulated cycle that allows cancer cells to rapidly adapt to the changing therapeutic pressures and microenvironments (121). This dynamism is reflected in the ability of lipid droplets to switch flexibly between “lipid storage” and “lipid mobilization” modes depending on cellular status. Under conditions of nutrient sufficiency or specific stresses (such as acidosis or drug treatment), cells promote lipid droplet accumulation by enhancing lipid synthesis or clearance pathways to store energy and sequester potentially toxic lipids (119, 122)—for example, endocrine-therapy-resistant ER+ breast cancer cells exhibit significant lipid metabolic reprogramming, characterized by elevated TAG levels and enhanced cytoplasmic lipid droplets, representing metabolic adaptation to therapeutic stress (10). When faced with energy shortages or the need for extensive membrane synthesis, cells initiate lipolysis and lipophagy pathways to break down lipid droplets and release fatty acids for β-oxidation or biosynthesis (113, 123). This cycle is precisely regulated by multiple signaling pathways. There is a profound interaction between autophagy and lipid droplet metabolism: Autophagy can provide precursors for lipid droplet synthesis, while selective autophagy (lipophagy) is an important pathway for lipid droplet degradation (123, 124). Endoplasmic reticulum stress and the ISR can also drive lipid droplet biogenesis as part of cellular adaptation to stress (125). Additionally, lipid droplets interact with organelles such as mitochondria and the endoplasmic reticulum through membrane contact sites, further integrating lipid metabolism with stress responses (126). In breast cancer, TM9SF1 drives the lipophagic flux through the AMPK–ULK1 signaling axis, promoting lipid droplet hydrolysis and FFA utilization to maintain energy homeostasis and metabolic adaptation in HER2+ breast cancer under nutrient deprivation (123). Therefore, the dynamic cycle of lipid droplets is a core manifestation of the metabolic plasticity of cancer cells, enabling them to maintain a survival advantage amidst fluctuations in metabolic demands caused by therapeutic pressure.

## 7 Mitochondrial fatty acid β-oxidation and other pathways

### 7.1 Activation of mitochondrial fatty acid β-oxidation

Endocrine-resistant cells exhibit upregulation of enzymes such as CPT1A/CPT1C, promoting the entry of long-chain fatty acids into the mitochondria for β-oxidation. Studies have confirmed that in ER+ breast cancer cells that have acquired endocrine therapy resistance, significant metabolic reprogramming occurs, one characteristic of which is the activation of the fatty acid β-oxidation pathway, a process highly dependent on CPT1 activity (11). This upregulation is not limited to CPT1A, and other carnitine transferases such as CPT1C may also be involved, collectively promoting mitochondrial transport of fatty acids—for example, in circulating tumor cells of melanoma, the expression of carnitine O-octanoyltransferase and carnitine acetyltransferase is upregulated. These enzymes regulate the transport of peroxisome-derived medium-chain fatty acids to the mitochondria, thereby providing fuel for mitochondrial fatty acid β-oxidation, which is crucial for tumor cell survival and metastasis (127). This dependence on the FAO pathway makes CPT1 family proteins key hubs connecting lipid uptake and mitochondrial energy metabolism.

In endocrine-resistant ER+ breast cancer cells, fatty acids are gradually broken down in the mitochondrial matrix via the β-oxidation process, with each cycle producing acetyl-CoA, NADH, and FADH2, thereby strongly driving the electron transport chain, enhancing OXPHOS, and ultimately generating large amounts of ATP (11) (128). This enhancement of energy metabolism is not an isolated event but works synergistically with the activation of AMPK signaling to jointly promote OXPHOS, providing sustained energy support for the survival and proliferation of resistant cancer cells under therapeutic pressure (11). Research indicates that fatty acid β-oxidation is an important energy source for cells under specific conditions (such as carbohydrate shortage), and the reducing equivalents it produces are crucial for maintaining electron transport chain function and ATP production (129). In tissues highly dependent on oxidative metabolism, such as the heart, fatty acid β-oxidation is central to maintaining normal function; in cancer cells, this pathway is hijacked to support their abnormal proliferation (130). Similarly, in breast cancer cells, enhanced OXPHOS, while meeting energy demands, may also be accompanied by the accumulation of metabolic byproducts.

The end product of fatty acid β-oxidation, acetyl-CoA, participates in multiple metabolic pathways, and its increased generation profoundly impacts the metabolism and epigenetic regulation of endocrine-resistant cells. First, as a key metabolite, acetyl-CoA can enter the TAC (tricarboxylic acid cycle) to be further oxidized, producing reducing equivalents that continuously support OXPHOS and ATP generation (128). This enhancement of metabolic flux underpins the maintenance of high cellular energy demands. However, the function of acetyl-CoA extends far beyond this; it is also the direct substrate for protein acetylation modifications, particularly histone acetylation. Therefore, excess acetyl-CoA produced by β-oxidation may be utilized by histone acetyltransferases, leading to epigenetic remodeling of chromatin structure (131). This modification can alter gene accessibility, thereby regulating a series of gene expression programs related to cell survival, proliferation, and drug resistance—for example, in studies of obesity-related metabolic disorders, nano-selenium-modified products affect metabolism by reducing the proportion of acetyl-CoA participating in the TAC while potentially altering its availability for other modification pathways (131). This suggests that the metabolic shunting of acetyl-CoA is a regulatable process. In the context of cancer, the expanded acetyl-CoA pool resulting from metabolic reprogramming likely provides abundant substrates for epigenetic modifications, thereby promoting a gene expression profile conducive to the development of drug resistance (132) (133).

### 7.2 Other pathways and the role of transcription factor

Peroxisomes are key organelles within cells responsible for oxidizing very-long-chain fatty acids and branched-chain fatty acids. Their metabolic activity plays a significant role in endocrine therapy resistance in ER+ breast cancer (10). Although studies directly investigating the association between peroxisomal activity and endocrine resistance are limited, the overall upregulation of lipid metabolism provides indirect evidence for enhanced peroxisomal function (134). When cancer cells adapt to endocrine therapy, they may enhance peroxisomal oxidation to obtain energy and biosynthetic precursors, thereby sustaining survival and proliferation. Furthermore, research has found that breast cancer risk in contralateral unaffected breast tissue is associated with a high expression of a set of lipid metabolism genes (135). This suggests that breast cancers of different ER subtypes may have distinct lipid metabolism patterns, and peroxisome-related pathways may be part of them. The observed increase in TAG storage and elevated PUFA levels in resistant cells may also be related to changes in peroxisomal processing capacity for specific fatty acids, such as very-long-chain fatty acids (10). A key byproduct of the peroxisomal fatty acid oxidation process is hydrogen peroxide, a reactive oxygen species with dual roles in cellular signaling: It can cause oxidative damage and also act as a second messenger to activate pro-survival pathways, thereby indirectly promoting endocrine therapy resistance (136). Increased hydrogen peroxide production due to enhanced peroxisomal activity may exceed the cell’s routine clearance capacity, establishing a sustained oxidative stress microenvironment (137). Enhanced peroxisomal FAO activity may be one of the metabolic adaptation strategies employed by breast cancer cells to cope with the pressure of endocrine therapy, warranting further in-depth research to elucidate its specific mechanisms and clinical significance.

The tumor microenvironment can activate a series of transcription factors, among which NF-κB is an important regulator of pro-survival and anti-apoptotic signaling pathways. Activation of NF-κB has been widely documented to be associated with cancer progression, therapy resistance, and poor prognosis—for instance, estrogen and its receptor signaling pathways can regulate various metabolic processes, including lipid metabolism, and metabolic changes, in turn, can affect the activity of signaling pathways (138). Additionally, selective estrogen receptor modulators such as tamoxifen and raloxifene, besides their anti-estrogenic effects on breast tissue, also impact lipid metabolism (139).

**Figure 1:**
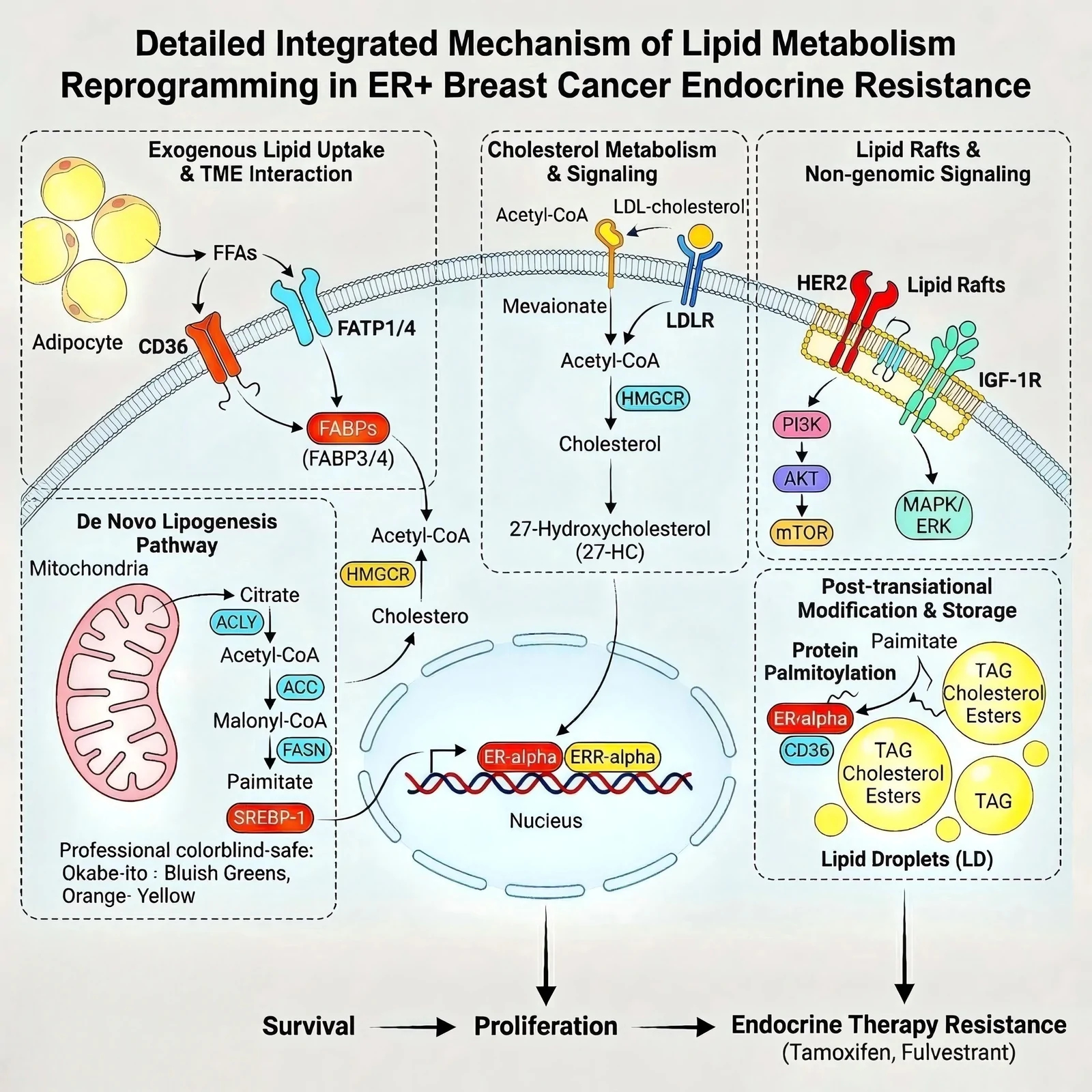
Detailed integrated mechanism of lipid metabolism reprogramming in ER+ breast cancer endocrine resistance. This image illustrates how lipid metabolism reprogramming leads to endocrine resistance in ER-positive breast cancer, detailing the metabolic and signaling events from lipid uptake to drug resistance. In the tumor microenvironment, adipocyte-derived free fatty acids are absorbed by tumor cells through CD36 and FATP1/4 and transported by FABPs. Simultaneously, *de novo* lipogenesis, controlled by SREBP-1 and enzymes like ACLY, ACC, and FASN, produces palmitate for membrane remodeling and metabolism. Cholesterol metabolism via the HMGCR pathway and LDLR-mediated uptake generates 27-HC, activating nuclear ERα and ERRα signaling. Lipid rafts enhance survival and growth signals through HER2 and IGF-1R, activating PI3K/AKT/mTOR and MAPK/ERK pathways. Palmitate supports ERα and CD36 stability via palmitoylation, while excess lipids are stored to prevent toxicity. These lipid metabolism pathways collectively boost tumor survival, proliferation, and resistance to endocrine therapies like tamoxifen and fulvestrant.

PPARs are members of the nuclear receptor superfamily and are key transcription factors regulating lipid metabolism, energy homeostasis, and inflammatory responses. They are activated through binding to ligands such as fatty acids and their derivatives, subsequently regulating the expression of a series of genes involved in fatty acid uptake, synthesis, storage, and oxidation. Research indicates that the expression of lipid metabolism genes is closely related to the ER status of breast cancer (135)—for example, in contralateral unaffected breast tissue, ER-negative cases exhibit an elevated expression of multiple lipid metabolism genes, including HMGCS2, and these genes may be regulated by transcription factors such as PPARs (140). PPARγ, an important subtype of the PPAR family, has garnered attention for its expression and activity. Some studies have explored the effects of different ratios of PUFAs on lipid metabolism genes and ER expression in MCF-7 breast cancer cells, finding that n-3 PUFAs can upregulate PPARγ expression (141). Since activation of PPARγ can influence lipid metabolism reprogramming, this may indirectly affect cancer cell sensitivity to TAM. Furthermore, endocrine resistance is associated with profound metabolic remodeling, with resistant cells exhibiting a shift toward TAG storage and accumulation of highly desaturated PUFAs (10). As core regulators of lipid metabolism, PPARs are likely involved in this adaptive change—for instance, PPARα primarily regulates FAO, and its enhanced activity may promote catabolism, including peroxisomal oxidation, thus providing energy for resistant cells. Concurrently, ESR1-activating mutations have been found to drive aberrant lipid biosynthesis and increase the expression of genes such as ACSL4 (long-chain acyl-CoA synthetase 4) (142). ACSL4 is a key enzyme for fatty acid activation, and its expression may also be regulated by PPARs (**Figure 1**).

## 8 Key upstream regulators of lipid metabolic reprogramming

### 8.1 Central role of transcription factor networks

SREBPs are core transcription factors that regulate cellular lipid homeostasis, and their aberrant activation plays a key role in endocrine therapy resistance in ER+ breast cancer. SREBPs drive lipid metabolic reprogramming in tumor cells by activating the expression of genes related to fatty acid and cholesterol synthesis, meeting the energy and biomembrane construction demands for their rapid proliferation (53). In obesity-induced breast cancer, dysregulation of the PI3K/AKT/mTOR pathway leads to the overactivation of SREBP-1, thereby promoting excessive lipid accumulation and creating a favorable microenvironment for tumor growth, metastasis, and drug resistance (53). This metabolic reprogramming is not limited to the context of obesity; in various cancers, the SREBP pathway is essential for supporting tumor growth, and the upregulation of its target genes is associated with poor patient prognosis (79). Specifically, the activity of SREBP is controlled by its key regulatory protein, SCAP (SREBP cleavage-activating protein). Activation of the SCAP/SREBP pathway is a crucial part of metabolic reprogramming in tumors such as pancreatic ductal adenocarcinoma, and inhibiting this pathway can prevent cancer cell growth under low-serum conditions due to insufficient lipid supply (79). In ER+ breast cancer, the oncogene AAMDC regulates the PI3K/AKT/mTOR signaling pathway, subsequently affecting the translation of ATF4 and MYC, ultimately modulating multiple metabolic processes including lipid metabolism. This provides an upstream signaling basis for the sustained activation of SREBP (143). Furthermore, under hypoxic conditions, triple-negative breast cancer cells promote cell survival by activating SREBP1-mediated lipogenesis, autophagy, and FAO, while inhibiting SREBP1 reduces ATP production and cell viability (144). Collectively, this evidence indicates that the sustained activation of SREBP driven by the PI3K/AKT/mTOR pathway is a key mechanism by which ER+ breast cancer overcomes endocrine therapy pressure and achieves metabolic adaptation and survival.

PPARγ and its coactivator peroxisome PGC-1α coordinate the expression of genes related to mitochondrial biogenesis and FAO. PPARγ, as a nuclear receptor transcription factor, has its activity regulated by ligands and is involved in the regulation of lipid metabolism and energy homeostasis. In the TME, particularly in breast cancers rich in adipocytes, FFAs from adipocytes can activate PPARα in tumor cells, thereby amplifying downstream metabolic effects (145)—for example, LXRs, another class of nuclear receptors regulating lipid and cholesterol metabolism genes, can be targeted by novel inverse agonists like GAC0001E5, which disrupt glutamine metabolism and redox homeostasis in breast cancer cells. This suggests that targeting nuclear receptor-mediated metabolic pathways is an effective strategy for intervention in breast cancer (146). Additionally, the regulation of metabolism by transcription factor networks is complex and interconnected. IRE1α is a key sensor of the UPR. By modulating the RIDD activity of IRE1α, it can reprogram lipid metabolism in cancer cells, for instance, by DGAT2 mRNA to affect TAG biosynthesis (120). This suggests that UPR (unfolded protein response)-related transcription factors such as XBP1, the spliced product of IRE1α, may also be associated with metabolic adaptation.

ER itself can also directly regulate a series of lipid metabolism genes. When ER signaling is blocked or mutated, its regulatory network becomes dysregulated, leading to metabolic reprogramming. ERα is not only a target of endocrine therapy but also a key transcription factor that can directly regulate the expression of a large number of downstream genes, including a series of genes involved in lipid metabolism. When the ER signaling pathway is blocked by drug treatment or when ESR1 acquires mutations, this finely tuned transcriptional regulatory network becomes severely dysregulated, driving tumor cells to undergo metabolic reprogramming to adapt to survival pressure and develop drug resistance (147). ESR1 mutations, particularly hotspot mutations in the ligand-binding domain (such as Y537S and D538G), result in constitutively active ERα variants that can exhibit transcriptional activity even in the absence of estrogen, leading to endocrine therapy resistance (148). Research has found that the key endoplasmic reticulum stress transcription factor XBP1 can synergize with ESR1 somatic mutants (point mutations and fusion mutations) to enhance their transactivation activity, thereby maintaining the expression of estrogen-regulated genes and promoting the growth of resistant cells (148). This indicates that ER mutants reshape their transcriptional regulatory network by recruiting different coregulators. Furthermore, the activity of ERα is precisely regulated by its protein stability, subcellular distribution, and post-translational modifications. Alterations in these mechanisms affect ERα’s ability to regulate downstream target genes, including metabolism-related genes, thereby influencing treatment response (149). When ER signaling is blocked by antiestrogen drugs such as FUL, tumor cells may activate alternative survival pathways through epigenetic reprogramming—for example, FUL treatment can induce the expression of VGLL1, a coactivator of the TEAD (transcriptional enhanced associate domain). VGLL1 then drives the expression of a series of genes that promote the growth of resistant cells, achieving transcriptional reprogramming (150). This reprogramming may indirectly affect metabolic pathways. More importantly, ERα can directly or indirectly regulate long non-coding RNAs (lncRNAs), and lncRNAs play a coordinating role in cancer metabolic reprogramming (151) —for instance, the estrogen-inducible lncRNA BNAT1 (breast cancer natural antisense transcript 1) can physically bind to the ERα protein, regulating ERα-dependent transcriptional regulation and playing an important role in endocrine-resistant cells (152). Therefore, abnormalities in the ER signaling pathway lead to the dysregulated expression of directly regulated lipid metabolism genes.

### 8.2 Epigenetics and post-transcriptional regulation

Epigenetic modifications play a key role in endocrine therapy resistance in ER+ breast cancer, with aberrant histone modifications as a crucial mechanism that drives metabolic reprogramming. Studies have shown that HDAC (histone deacetylase) inhibitors can affect a wide range of gene expression patterns, including lipid metabolism-related genes, by altering chromatin states, thereby interfering with cancer cell adaptability (2)—for instance, in ER+ breast cancer, epigenetic reprogramming, particularly post-translational modifications of histones, is closely associated with the development of endocrine therapy resistance (2). Specifically, HDAC inhibitors such as LBH589 can reverse transcriptional repression caused by HDAC overexpression, thereby restoring the normal regulation of specific signaling pathways implicated in cancer progression (153). This epigenetic intervention not only affects proliferation-related genes but also profoundly alters the metabolic state of cells. Research has found that in ER+ breast cancer, epigenetic changes, including DNA methylation and histone modifications, can lead to the silencing or aberrant activation of key genes, thereby promoting tumor progression and resistance to therapy (3). The combined application of HDAC inhibitors and AI has shown potential to overcome resistance in preclinical and clinical studies. A meta-analysis confirmed that in patients with hormone receptor-positive advanced breast cancer who had progressed on prior endocrine therapy, the combination of an HDAC inhibitor with an AI significantly prolonged progression-free survival and improved the objective response rate compared to AI alone (154). This suggests that pharmacological intervention in histone acetylation status can reshape the transcriptional program of tumor cells, which may include the regulation of key enzymes involved in lipid synthesis and catabolism. Furthermore, epigenetic therapies can induce genome-wide DNA hypomethylation, resulting in extensive three-dimensional epigenomic dysregulation characterized by decompaction of higher-order chromatin structures and compromised insulation at topologically associating domain boundaries (155). Therefore, epigenetic therapies targeting HDACs, by remodeling chromatin, represent a promising strategy for reversing endocrine resistance mechanisms, including lipid metabolic reprogramming.

Non-coding RNAs, particularly microRNAs, play a fine-tuning role in lipid metabolic reprogramming and endocrine therapy resistance in ER+ breast cancer as key factors in post-transcriptional regulation. These short RNA molecules can affect the expression of various proteins, including lipid metabolic enzymes, by binding to the 3’ UTR of target gene mRNAs, leading to their degradation or translational inhibition (156). Studies have found that specific miRNAs, such as miR-22, can directly regulate the expression of ERα—for example, treatment of MCF7 cells with the long-chain fatty acid oleic acid upregulates miR-22 and miR-221, which have ER-inhibitory effects, leading to a reduction in ERα mRNA and nuclear protein levels, thereby impairing the cell’s response to estradiol and TAM (157). Beyond directly targeting ER, miRNAs are also involved in regulating downstream metabolic pathways. Long non-coding RNAs play multiple roles in mediating tamoxifen resistance, with mechanisms including enhancing ER signaling, inhibiting apoptosis, regulating autophagy, and acting as competitive endogenous RNAs (158). Notably, the mediator complex subunit MED1 can regulate the transcription of the ER-dependent miR-191/425 cluster, promoting breast cancer cell proliferation and migration (156). This suggests a close interplay between transcriptional and post-transcriptional regulation, jointly influencing tumor phenotypes. At the metabolic level, neurofibromin deficiency drives metabolic reprogramming in ER+ breast cancer, characterized by a significant expansion of lipid pools (especially TAG) (159). Therefore, non-coding RNAs, exemplified by miR-22, by targeting ERα or potentially metabolic enzyme genes, constitute a key bridge connecting the epigenetic environment, metabolic state, and endocrine therapy response, providing direction for the development of new biomarkers and therapeutic targets.

RNA-binding proteins can precisely regulate lipid metabolic flux by modulating the stability and translation efficiency of metabolic enzyme mRNAs. In ER+ breast cancer, specific RNA-binding proteins have been shown to regulate the expression of genes related to cell growth and metabolism, thereby affecting tumor progression and therapeutic response. Studies indicate that PSPC1 interacts with another RNA-binding protein, PSF, and together they participate in the post-transcriptional regulation of the ESR1 and SCFD2 genes (160). A high expression of PSPC1 correlates with poor prognosis in ER+ breast cancer patients, and activation of the PSPC1/PSF/SCFD2 axis is a strong prognostic factor (160). Importantly, SCFD2 downstream targets include genes involved in cell cycle regulation and survival, and silencing SCFD2 can inhibit the *in vivo* growth of TAM-resistant breast tumors (160). This reveals a pathway through which RNA-binding proteins indirectly affect tumor cell metabolic adaptability and resistant phenotypes by regulating specific mRNAs. Another example is the oncogene AAMDC, which regulates the translation process of key transcription factors such as ATF4 and MYC by controlling the PI3K/AKT/mTOR signaling pathway. This regulation thereby modulates the transcriptional activity of various metabolic enzymes, including those involved in lipid metabolism (143). Overexpression of AAMDC is sufficient to activate AKT signaling, leading to estrogen-independent tumor growth, and its high expression is associated with sensitivity to PI3K-mTOR inhibitors (143). This highlights the importance of translational-level regulation in integrating growth signals with metabolic reprogramming. Additionally, the development of endocrine therapy resistance may involve non-genetic cellular state transitions, such as entering a dormant state through epigenetic reprogramming (161). In such adaptive processes, RNA-binding proteins may maintain the low metabolic state of dormant cells and promote “awakening” when conditions are favorable by dynamically regulating the translation and turnover of metabolism-related mRNAs. Therefore, as executors of the post-transcriptional regulatory network, RNA-binding proteins can rapidly respond to intracellular and extracellular signals (such as growth factors, nutrient status, and therapeutic pressure) by precisely adjusting the synthesis rates of lipid metabolic enzymes like ACLY and FASN as well as other key proteins. This directly controls lipid metabolic flux and ultimately affects the survival, proliferation, and response to endocrine therapy of breast cancer cells (**Table 2**).

**Table 2:** Endocrine resistance in ER+ breast cancer: Molecular targets and advances in drug development.

Target	Therapeutic Strategy	Mechanism of Action	Status in Breast Cancer	Refs.
FASN	TVB-2640 (Denifanstat), Orlistat	Inhibits the terminal step of de novo palmitate synthesis.	Restores sensitivity to Tamoxifen and Fulvestrant; TVB-2640 in clinical trials.	(10, 43)
ACC	ND-646, Firsocostat	Blocks the rate-limiting step of fatty acid synthesis.	Synergistic with endocrine therapy; inhibits metastasis in resistance models.	(108)
CD36	SSO, Neutralizing antibodies	Inhibits uptake of exogenous long-chain fatty acids.	Partially reverses resistance; blocks lipid-mediated Src activation and metastasis.	(60, 62)
HMGCR	Statins (e.g., Atorvastatin)	Inhibits the mevalonate pathway (cholesterol synthesis).	Window-of-opportunity trials show antiproliferative effects in primary tumors.	(86, 87)
CPT1/FAO	Etomoxir, Perhexiline	Inhibits mitochondrial entry of fatty acids for oxidation.	Sensitizes resistant cells to therapy-induced apoptosis; reduces energy availability.	(74, 180)
ACLY	NDI-091143, BMS-303141	Inhibits citrate conversion to acetyl-CoA.	Promotes apoptosis in resistant cells by depleting the acetyl-CoA pool.	(74)

The table integrates six critical elements: It identifies target names (e.g., CD36 and FASN) and their associated signaling pathways (e.g., lipid uptake and *de novo* lipogenesis), contrasts target expression levels between drug-resistant and sensitive cell or tissue groups, and classifies clinical drug types (e.g., inhibitors and antagonists). Furthermore, it details the developmental stage of each drug, ranging from preclinical studies to phase II clinical trials, and provides references to relevant literature, thereby serving as a valuable resource for prioritizing targets and advancing research on anti-resistance therapies.

## 9 Therapeutic strategies targeting lipid metabolism to overcome endocrine resistance

### 9.1 Clinical applications of drugs targeting lipid metabolism

FASN inhibitors such as TVB-2640 have entered clinical trials and, when combined with TAM or AI, have shown potential to reverse resistance in preclinical models and early clinical trials (162). In breast cancer, particularly the ER+ subtype, aberrant upregulation of FASN supports rapid tumor cell proliferation, membrane biosynthesis, and resistance to therapies, including endocrine therapy (162). Preclinical studies indicate that inhibiting FASN disrupts lipid biosynthesis, which leads to the accumulation of metabolic intermediates, induces metabolic stress, and triggers cell cycle arrest or apoptosis in cancer cells (162). TVB-2640, also known as denifanstat, is a first-in-class selective FASN inhibitor with favorable pharmacokinetic properties. It has demonstrated potent antitumor activity in preclinical models (162). Importantly, FASN blockade not only impairs tumor growth but also enhances the efficacy of existing therapies, including chemotherapy, endocrine therapy, and targeted therapy, supporting combination strategies (162)—for example, in ER+ breast cancer, the efficacy of TAM is partly mediated through the modulation of key lipid metabolism genes such as FASN (163). Clinical evidence suggests that such combination regimens may overcome resistance by synergistically disrupting energy supply and the membrane structural integrity of tumor cells (162). However, the translation of FASN inhibitors into clinical use is notably restricted by their limited efficacy as standalone treatments, frequent significant off-target effects, poor oral absorption, and intolerable toxic side effects, such as gastrointestinal problems (162). The regulatory effects of FASN inhibition on immune components within the tumor microenvironment, which depend on the context, are major challenges to its clinical use and treatment optimization (162).

ACC inhibitors such as ND-646, by inhibiting the first step of fatty acid synthesis, effectively suppress the growth of resistant tumors and may enhance chemotherapy sensitivity. Inhibiting ACC fundamentally blocks the *de novo* fatty acid synthesis pathway, thereby affecting tumor cell energy storage, membrane construction, and signaling. In breast cancer, lipid metabolism reprogramming is a core feature of tumor progression and resistance (164). ACC, as the entry point of this pathway, represents an attractive therapeutic target. Studies have shown that the ACC inhibitor ND-646 effectively blocks fatty acid synthesis in resistant tumor cells by inhibiting ACC activity (165). This inhibition leads to the depletion of intracellular lipid precursors, triggering a metabolic crisis and thereby suppressing tumor cell growth and proliferation (165). In preclinical models, ACC inhibition not only suppresses tumor growth as a monotherapy but may also enhance the sensitivity of chemotherapy drugs. The mechanism may involve ACC inhibition depriving tumor cells of the lipid resources needed under chemotherapy pressure to repair damaged cell membranes or generate survival signals, making tumor cells more vulnerable to chemotherapy-induced cell death (164). Additionally, alterations in lipid metabolism are closely related to immune responses in the TME (164). ACC inhibition may indirectly enhance therapeutic responses by altering the lipid composition of tumor cells and affecting their interactions with immune cells. Although clinical research data on direct ACC inhibitors in the context of endocrine therapy resistance in breast cancer are relatively limited, based on its central role in lipid synthesis and the efficacy demonstrated in other cancer models, targeting ACC (e.g., using ND-646) offers a promising new direction for overcoming endocrine therapy resistance in ER+ breast cancer. Its combination with chemotherapy or other targeted therapies warrants further exploration.

CPT1A inhibitors, such as etomoxir, block fatty acid oxidation, leading to intracellular lipid accumulation and an energy crisis that selectively kills resistant cells dependent on fatty acid oxidation. CPT1A is the rate-limiting enzyme of mitochondrial FAO, responsible for transporting long-chain fatty acids into the mitochondrial matrix for oxidative breakdown to produce energy. In tumor cells, especially those under metabolic stress (e.g., hypoxia, nutrient deficiency, or drug treatment), FAO is often upregulated as an important alternative pathway to maintain energy homeostasis and survival (166). Studies on esophageal squamous cell carcinoma have demonstrated that STING suppresses FAO by promoting the ubiquitination and degradation of CPT1A, thereby inhibiting tumor progression (166). This suggests that CPT1A-mediated FAO plays a critical role in tumor growth. In breast cancer, particularly the ER+ subtype that may develop resistance to endocrine therapy, tumor cells may enhance FAO to adapt to therapeutic pressure and survive. CPT1A inhibitors such as etomoxir irreversibly block the FAO process by inhibiting CPT1A (166). This blockade leads to two main consequences. First, cells cannot utilize fatty acids to produce sufficient ATP, triggering an energy crisis. Second, unoxidized fatty acids accumulate intracellularly, potentially causing lipotoxicity, including lipid peroxidation and organelle dysfunction (166). Resistant cells dependent on FAO are particularly sensitive to energy deprivation and lipotoxicity, which allows CPT1A inhibitors to selectively induce cell death in these cells. Studies have shown that in tumor cells with reduced STING expression, FAO is enhanced, and treatment with etomoxir can reverse this pro-growth effect (166). In breast cancer, lipid metabolism reprogramming is closely associated with therapy resistance (164). Therefore, targeting CPT1A to inhibit FAO may be an effective strategy for overcoming endocrine therapy resistance in ER+ breast cancer. By depriving resistant cells of their energy source and inducing metabolic stress, CPT1A inhibitors such as etomoxir hold promise for combination with endocrine therapy drugs to synergistically kill resistant cells, offering a new therapeutic window for reversing endocrine therapy resistance.

### 9.2 Interfering with key nodes of lipid metabolism regulation

The use of mTORC1 inhibitors such as everolimus can reduce SREBP activity, thereby inhibiting the synthesis of fatty acids and cholesterol. This combination strategy has been approved by the FDA for advanced ER-positive breast cancer. mTORC1 is a central hub that senses nutrient and growth factor signals and coordinates cell growth and metabolism (167). In ER+ breast cancer, hyperactivation of the mTORC1 signaling pathway is one of the important drivers of endocrine therapy resistance. mTORC1 profoundly affects the reprogramming of lipid metabolism by regulating downstream effector molecules, particularly by activating SREBP to promote the *de novo* synthesis of fatty acids and cholesterol (168). SREBP, as a key transcription factor for lipid synthesis, is strictly regulated by mTORC1. When mTORC1 is activated, it promotes the maturation and nuclear translocation of SREBP, thereby upregulating the expression of key lipid synthesis enzymes such as FASN and ACC. This drives lipid accumulation in tumor cells to meet their energy and membrane structure demands for rapid proliferation (168). Therefore, targeting mTORC1 has become an important strategy to reverse lipid metabolic reprogramming and overcome drug resistance. Everolimus, as an mTORC1 inhibitor, can effectively downregulate the transcriptional activity of SREBP by inhibiting the activity of this complex, thereby suppressing the fatty acid and cholesterol synthesis pathways (167). Preclinical and clinical studies have confirmed that the combination of everolimus with endocrine therapy drugs can significantly reverse or delay endocrine therapy resistance in patients with advanced ER+ breast cancer and improve progression-free survival. Based on its clear efficacy, this combination therapy regimen has been approved by the US Food and Drug Administration and has become one of the standard strategies for treating advanced ER+ breast cancer (167). This successful example not only validates the central role of the mTORC1–SREBP axis in breast cancer lipid metabolic reprogramming but also provides a paradigm for overcoming therapeutic resistance by interfering with key metabolic nodes.

The development of LXR agonists aims to induce cholesterol efflux and inhibit SREBP activity, but their impact on systemic lipid metabolism requires optimization via tissue-selective agents. LXR is a member of the nuclear receptor superfamily and functions dually as a “sensor” and “regulator” in cholesterol homeostasis (168). In tumor cells, activating LXR can induce the expression of cholesterol efflux proteins such as ABCA1, promoting the reverse transport of intracellular cholesterol and thereby reducing intracellular cholesterol load (169). More importantly, activation of LXR can interfere with the processing and maturation of SREBP, inhibiting its transcriptional activity and subsequently reducing the *de novo* synthesis of fatty acids and cholesterol at its origin (168). This dual mechanism of action (simultaneously promoting cholesterol clearance and inhibiting lipid synthesis) makes LXR an attractive target for intervening in breast cancer lipid metabolic reprogramming and overcoming endocrine therapy resistance. However, the clinical translation of LXR agonists faces significant challenges, primarily due to their broad impact on systemic lipid metabolism. In tissues such as the liver, although LXR activation is beneficial for cholesterol efflux, it can also induce the expression of genes such as FASN, potentially leading to adverse metabolic side effects like hypertriglyceridemia and hepatic steatosis (168). This tissue-nonselective effect limits the clinical application of first-generation LXR agonists. Therefore, the current research focus is on developing tissue-selective or function-selective LXR modulators—for example, efforts are aimed at designing compounds that can selectively activate LXR in tumor tissues while avoiding strong activation of lipid synthesis programs in the liver. Additionally, exploring the crosstalk between LXR and other signaling pathways (such as AMPK) or developing ligands targeting specific LXR subtypes (LXRα or LXRβ) are also strategies to optimize their therapeutic window (168). Through innovations in medicinal chemistry and delivery systems, the targeted delivery of LXR agonists to tumor sites represents a key direction for overcoming their systemic side effects. This approach will help realize their potential in anti-tumor metabolic reprogramming in the future.

Targeting CD36 with monoclonal antibodies or small-molecule inhibitors aims to block the exogenous lipid supply to cancer cells and is currently under preclinical investigation (170). Currently, monoclonal antibodies and small molecule inhibitors targeting CD36 are in active preclinical research stages. These interventions, by blocking the fatty acid transport function of CD36, can significantly reduce the uptake of extracellular lipids by breast cancer cells, leading to intracellular lipid depletion, energy stress, and ultimately inhibiting the proliferation, invasion, and metastatic capabilities of tumor cells (170). Studies have shown that inhibition of CD36 can produce synergistic effects with existing therapies—for example, in the TME, reprogramming of lipid metabolism profoundly affects the function of immune cells. The survival and function of immunosuppressive cells such as MDSCs depend on specific lipid metabolic pathways. Targeting CD36 may indirectly regulate anti-tumor immune responses by altering lipid availability in the microenvironment (171). However, developing effective CD36 inhibitors also faces challenges. CD36 is expressed in various normal tissues (such as muscle, adipose tissue, and immune cells), and systemic inhibition may lead to off-target effects on metabolism or immune function. Additionally, cancer cells may compensate for the loss of CD36 function by upregulating other lipid uptake pathways (such as FABPs) or enhancing endogenous lipid synthesis, thereby developing resistance (164). Future research needs to deeply explore the function of CD36 in specific breast cancer subtypes, evaluate its safety and efficacy as a therapeutic target, and consider combining it with other drugs targeting lipid synthesis or oxidation to overcome metabolic compensation mechanisms.

### 9.3 Synthetic lethality strategy based on metabolic vulnerability

The dual-hit strategy of simultaneously inhibiting fatty acid synthesis and oxidation effectively blocks the compensatory pathways of lipid metabolism, thereby triggering a severe metabolic crisis and inducing cell death. In tumor cells, the lipid metabolic network exhibits high plasticity and redundancy. Single-target strategies against fatty acid synthesis or oxidation are often limited in efficacy due to the compensatory activation of metabolic pathways. Therefore, the “dual-hit” strategy, which simultaneously inhibits these two critical branches, aims to completely cut off the lipid supply to tumor cells, inducing irreversible metabolic crisis. This synthetic lethality strategy has been validated in various cancer models (172)—for example, in pancreatic ductal adenocarcinoma, targeting the lipid kinase PIKfyve, which is essential for lysosomal function, forces tumor cells to upregulate *de novo* lipid synthesis programs, thereby creating dependency on KRAS–MAPK signaling-driven lipid synthesis (173). Inhibiting PIKfyve and the KRAS–MAPK pathway together can synergistically eliminate the tumor burden in PDAC, demonstrating the effectiveness of disrupting lipid homeostasis and synthesis pathways (173). Similarly, in colorectal cancer, inhibiting FASN reduces DNA damage repair capacity. When combined with the chemotherapeutic drug irinotecan, it synergistically enhances DNA double-strand breaks and apoptosis and delays tumor recurrence (174). These studies indicate that when one lipid metabolic pathway is inhibited, tumor cells become highly reliant on another compensatory pathway to survive. By designing drug combinations that simultaneously block *de novo* fatty acid synthesis (e.g., targeting FASN or ACC1) and subsequent β-oxidation or storage processes, the sources of energy and membrane components in tumor cells can be effectively disrupted, leading to lipid depletion, endoplasmic reticulum stress, and reactive oxygen species accumulation, ultimately triggering severe metabolic cell death. This strategy is particularly suitable for tumors already under metabolic stress due to oncogenic signals (e.g., KRAS mutations) or microenvironmental pressures (e.g., nutrient deprivation), offering new insights for overcoming endocrine therapy resistance (175).

Combining lipid metabolism inhibitors with drugs that induce ferroptosis may result in synergistic effects because the reprogramming of lipid metabolism alters cellular sensitivity to lipid peroxidation. The reprogramming of lipid metabolism in tumor cells, including the enrichment of PUFAs and alterations in the antioxidant defense system, directly determines their sensitivity to ferroptosis (176). Therefore, combining drugs targeting lipid metabolism with ferroptosis inducers is expected to produce powerful synergistic killing effects. The core of this synthetic lethality strategy is that lipid metabolism inhibitors can alter the lipid composition of tumor cells, making them more prone to lipid peroxidation, thereby “sensitizing” tumor cells to ferroptosis inducers (177). Research has confirmed that in AML (acute myeloid leukemia), Aldh3a2 (cancer cell dependence on aldehyde dehydrogenase 3a2) clears long-chain fatty aldehydes produced by lipid peroxidation to resist oxidative damage and ferroptosis (178). Inhibiting Aldh3a2 alone has limited effects on AML cells, but when combined with GPX4 (glutathione peroxidase 4, a key ferroptosis inhibitory protein) inhibitors, it exhibits significant synthetic lethality effects (178). This indicates that simultaneously disrupting the clearance system for lipid peroxidation products (Aldh3a2) and the core antioxidant defense system (GPX4) can synergistically induce lethal lipid peroxidation accumulation. In ER+ breast cancer, endocrine therapy resistance is often accompanied by profound remodeling of lipid metabolism, which may alter the cell’s defense capacity against lipid peroxidation—for example, increased FASN activity may change the saturation degree of membrane lipids, while abnormalities in cholesterol metabolism may affect membrane fluidity. Targeting these specific lipid metabolism targets, such as using FASN inhibitors, may increase the proportion of PUFAs in the cell membrane or deplete protective antioxidants (e.g., glutathione), thereby creating favorable conditions for ferroptosis inducers (e.g., GPX4 inhibitors, erastin, or RSL3). This combination strategy based on metabolic vulnerability offers a highly promising new direction for overcoming therapy resistance driven by lipid metabolism reprogramming (179).

**Figure 2:**
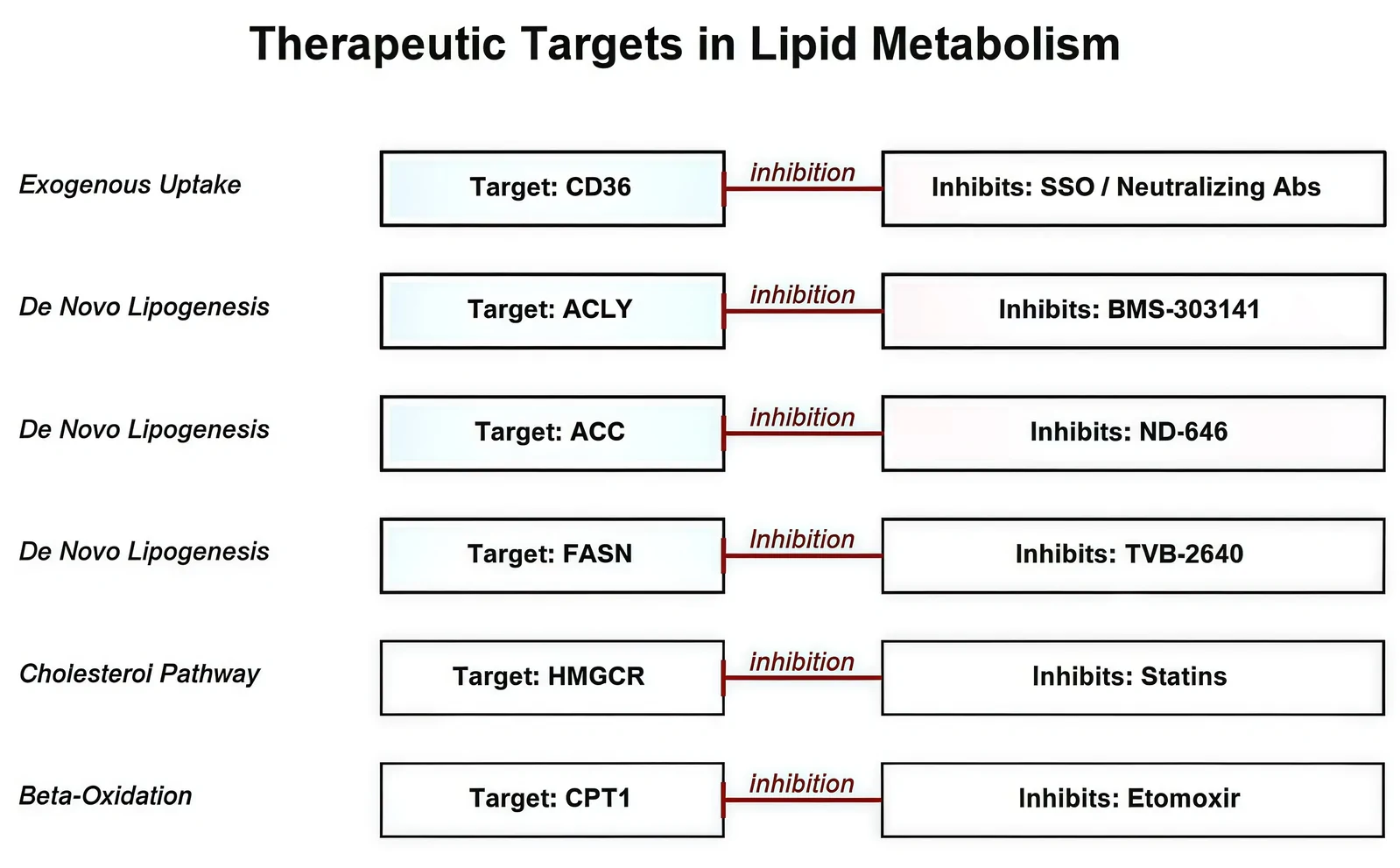
Therapeutic targets in lipid metabolism. This image systematically categorizes the principal therapeutic targets within dysregulated lipid metabolism pathways, along with their corresponding specific inhibitory agents, which are engineered to address the lipid metabolic reprogramming that underlies endocrine resistance in breast cancer. Targets in lipid metabolism are organized by pathways. In the exogenous lipid uptake pathway, CD36 is the main target, inhibited by SSO or neutralizing antibodies. For *de novo* lipogenesis, key targets are ACLY, ACC, and FASN, inhibited by BMS-303141, ND-646, and TVB-2640, respectively. In cholesterol synthesis, HMGCR is targeted by statins. In mitochondrial fatty acid beta-oxidation, CPT1 is targeted by etomoxir. This overview of targets and inhibitors aids in developing combination strategies to overcome endocrine therapy resistance in breast cancer.

Tumor heterogeneity determines the diversity of metabolic phenotypes. Therefore, patient stratification based on tumor-specific lipid metabolism gene expression profiles or metabolite characteristics is a prerequisite for achieving precise and efficient metabolic-targeted therapy. By integrating multi-omics technologies (e.g., transcriptomics and metabolomics) and advanced metabolic imaging, tumor subpopulations that are sensitive to specific lipid metabolism inhibitors can be identified—for example, in TNBC, studies have revealed different metabolic dependencies. TNBC subtypes overly reliant on OXPHOS are sensitive to the OXPHOS inhibitor IACS-10759, and their sensitivity is associated with the high expression of mitochondrial genes (180). Further *in vivo* functional genomics screening found that such tumors exhibit synthetic lethality effects with the combination therapy of IACS-10759 and the CDK4/6 inhibitor palbociclib (180). This suggests that OXPHOS metabolic features can be used as biomarkers to screen patients who may benefit from this combination therapy. Another example is that in breast cancer, tumor cells with high IDH2 (isocitrate dehydrogenase 2) expression heavily depend on the serine biosynthesis pathway for survival, making PHGDH (3-phosphoglycerate dehydrogenase) a synthetic dosage lethality target (181). Clinical data analysis shows that breast cancer patients with high IDH2 but low PHGDH expression have longer survival, while patients with high IDH2 and high PHGDH may benefit from PHGDH inhibitor treatment (181). This provides direct evidence for patient stratification based on IDH2 and PHGDH expression profiles. For ER+ breast cancer, future research needs to systematically delineate the dynamic changes in lipid metabolism gene expression and metabolite profiles of tumors before and after endocrine therapy resistance, identifying specific expression patterns of key enzymes such as FASN, ACC1, CPT1A, or accumulation characteristics of specific lipid species (e.g., certain phospholipids and sphingolipids). These features can serve as biomarkers to predict patient responses to fatty acid synthesis inhibitors, fatty acid oxidation inhibitors, or ferroptosis inducers, thereby guiding clinical decisions and enabling truly individualized metabolic-targeted therapy (175) (**Figure 2**).

## 10 Conclusions and perspectives

Lipid metabolic reprogramming is one of the core mechanisms by which ER+ breast cancer cells adapt to endocrine therapy pressure and acquire drug resistance. This process involves coordinated changes at multiple levels, such as fatty acid synthesis, uptake, storage, oxidation, and cholesterol metabolism. Regarding fatty acid synthesis, endocrine-resistant cells exhibit enhanced FASN activity, which has been confirmed by 13C2-acetate tracer experiments; moreover, the FASN inhibitor TVB-2640 can effectively reduce this activity. Regarding fatty acid oxidation, the splicing variant BQ323636.1 of the NCOR2 gene upregulates the expression of ACSL4 by disrupting the interaction between NCOR2 and PPARγ, which enhances FAO and increases the production of acetyl-CoA and ATP, thereby providing energy for tumor proliferation. Regarding cholesterol metabolism, clinical research data indicate that serum lipid levels are closely related to endocrine resistance. In ER+ breast cancer patients receiving endocrine therapy, the average serum TG and LDL-C levels throughout the progression-free survival period under endocrine therapy are independent predictors of endocrine resistance. In HER2+/ER+ breast cancer, cells that acquire endocrine resistance also exhibit upregulated lipid metabolism and an elevated expression of ferroptosis-related antioxidant genes (such as GPX4), further linking alterations in lipid metabolism to the remodeling of the cellular antioxidant defense system.

Lipid metabolic reprogramming is tightly regulated by a complex network that includes SREBP, PPARγ, ER itself, and its downstream signaling pathways and also interacts with epigenetic and microenvironmental factors. SREBP and PPARγ are core transcription factors regulating lipid synthesis and storage. Studies have shown that in pathological states of lipid metabolism disorders, such as in dairy cow fatty liver models, the protein abundance of SREBP-1c and its downstream target genes (such as ACC1 and FASN) significantly increases, directly driving *de novo* fatty acid synthesis. Similarly, the upregulation of PPARγ expression is closely related to the increase in lipid synthesis proteins (such as ACC1), collectively promoting abnormal lipid accumulation within cells. These findings suggest that in breast cancer cells, abnormal activation of SREBP and PPARγ may enhance fatty acid synthesis, providing energy and membrane structural components necessary for the survival and proliferation of resistant cells, thereby countering the pressure of endocrine therapy.

Targeting key enzymes or regulatory nodes in lipid metabolism, such as FASN, ACC, CPT1A, or mTOR, shows great therapeutic potential for overcoming endocrine resistance, and related drugs have entered different stages of clinical development. The clinically relevant FASN inhibitor TVB-2640 effectively reduces FASN activity and lipid storage in endocrine-resistant cells in preclinical studies, although its inhibitory effect on cell growth varies among different cell lines, suggesting heterogeneity in the metabolic dependence of resistant cells. Notably, the levels of highly desaturated PUFAs are elevated in resistant cells, and TVB-2640 has a limited impact on these levels, indicating that targeting specific lipid-metabolism-dependent pathways, especially the PUFA generation pathway, may be an effective strategy to overcome resistance. Additionally, ACC is another key enzyme in fatty acid synthesis, and its synergistic upregulation with FASN has been widely observed in endocrine resistance and metabolic-related diseases. Inhibiting ACC can reduce the substrates for fatty acid synthesis, thereby interfering with the energy supply and membrane synthesis of tumor cells. CPT1A is the rate-limiting enzyme in mitochondrial fatty acid β-oxidation, and its dysfunction is associated with various metabolic diseases and cancers. In the context of lipid metabolism disorders, such as in metabolic dysfunction-associated steatotic liver disease, the expression of CPT1A is often downregulated, leading to impaired fatty acid oxidation and lipid accumulation. Significant progress has been made in developing drugs targeting these enzymes. In addition to TVB-2640 and the marketed mTOR inhibitors, other drugs targeting lipid metabolism, such as CPT1A inhibitors, are in different stages of clinical development.

Future research should thoroughly analyze the specific lipid metabolic characteristics of endocrine therapy-resistant subtypes in ER+ breast cancer. Tumor heterogeneity, including molecular subtypes and functional compartments of the TME, is key to causing resistance and differences in efficacy—for example, in ER+ breast cancer, the ESR1 mutation subtype exhibits unique lipid metabolic reprogramming, driving abnormal lipid biosynthesis and uptake, accompanied by the upregulation of ACSL4 expression, making it sensitive to ferroptosis inducers. Conversely, other resistant subtypes may rely on different pathways such as cholesterol synthesis or sphingolipid metabolism. Therefore, the use of technologies such as single-cell sequencing, spatial transcriptomics, and multi-omics analysis to systematically characterize the lipid metabolic profiles of different resistant subtypes (such as ESR1 mutant, PIK3CA mutant, FGFR amplified, and others) will help identify core metabolic nodes driving specific resistant phenotypes, laying the foundation for precise intervention. Simultaneously, stromal cells such as cancer-associated fibroblasts in the TME also influence chemotherapy and endocrine therapy responses through pathways like lipid metabolism, and analyzing these intercellular communication networks is crucial for a comprehensive understanding of resistance mechanisms.

## Glossary

27-HC: 27-hydroxycholesterol
ABCA1/G1: ATP-binding cassette transporter A1/G1
ACC: acetyl-CoA carboxylase
ACLY: ATP-citrate lyase
ACSL4: long-chain acyl-CoA synthetase 4
ACSS3: acyl-CoA synthetase short-chain family member 3
AI: aromatase inhibitor
AKT: protein kinase B
Aldh3a2: cancer cell dependence on aldehyde dehydrogenase 3a2
AML: acute myeloid leukemia
ATGL: adipose triglyceride lipase
BNAT1: breast cancer natural antisense transcript 1
CDK4/6: cyclin-dependent kinase 4/6
CH25H: cholesterol 25-hydroxylase
CHK: checkpoint kinase
CPT1A/C: carnitine palmitoyltransferase 1A/C
CRL4B: Cullin4B–RING ubiquitin ligase complex
DGAT: diacylglycerol acyltransferase
DHCR24: 24-dehydrocholesterol reductase
EGFR: epidermal growth factor receptor
EMT: epithelial–mesenchymal transition
ER+: estrogen receptor positive
ERK1/2: extracellular signal-regulated kinase 1/2
FABP: fatty acid-binding protein
FAO: fatty acid oxidation
FASN: fatty acid synthase
FATP: fatty acid transport protein
FFA: free fatty acid
FUL: fulvestrant
GPX4: glutathione peroxidase 4
GRP78: glucose-regulated protein 78
HDAC: histone deacetylase
HER2: human epidermal growth factor receptor 2
HMGCR: 3-hydroxy-3-methylglutaryl-CoA reductase
HRG: autocrine heregulin
IDH2: isocitrate dehydrogenase 2
IGF-1R: insulin-like growth factor-1 receptor
IGFBP6: insulin-like growth factor-binding protein 6
INSIG1/2: insulin-induced gene 1/2
KDM5B: lysine demethylase 5B
LDLR: low-density lipoprotein receptor
LET: letrozole
LXR: liver X receptor
MAPK: mitogen-activated protein kinase
mTOR: mammalian target of rapamycin
NUCB2: nucleobindin 2
OHT: 4-hydroxytamoxifen
OXPHOS: oxidative phosphorylation
PCSK9: proprotein convertase subtilisin/kexin type 9
PFKL: phosphofructokinase, liver type
PHGDH: 3-phosphoglycerate dehydrogenase.
PI3K: phosphatidylinositol 3-kinase
PLIN2/3: perilipin 2/3
PPAR: peroxisome proliferator-activated receptor
PUFA: polyunsaturated fatty acid
RIDD: regulated IRE1-dependent decay
ROS: reactive oxygen species
SCAP: SREBP cleavage-activating protein
SERD: selective estrogen receptor degrader
SERM: selective estrogen receptor modulator
SREBP: sterol regulatory element-binding protein
TAC: tricarboxylic acid cycle
TAG: triglyceride
TAM: tamoxifen
TEAD: transcriptional enhanced associate domain
TME: tumor microenvironment
TNBC: triple-negative breast cancer
UPR: unfolded protein response

## References

[1] Lu Y Liu W . Selective estrogen receptor degraders (SERDs): a promising strategy for estrogen receptor positive endocrine-resistant breast cancer. J Med Chem. (2020) 63:15094–114. doi: 10.1021/acs.jmedchem.0c00913 33138369

[2] Clusan L Ferrière F Flouriot G Pakdel F . A basic review on estrogen receptor signaling pathways in breast cancer. Int J Mol Sci. (2023) 24:6834. doi: 10.1016/j.canlet.2021.05.030 37047814 PMC10095386

[3] Gao Y Yu Y Zhang M Yu W Kang L . Mechanisms of endocrine resistance in hormone receptor-positive breast cancer. Front Oncol. (2024) 14:1448687. doi: 10.3389/fonc.2024.1448687 39544302 PMC11560879

[4] Palaniappan M . Current therapeutic opportunities for estrogen receptor mutant breast cancer. Biomedicines. (2024) 12:2700. doi: 10.3390/biomedicines12122700 39767607 PMC11673253

[5] Brett JO Spring LM Bardia A Wander SA . ESR1 mutation as an emerging clinical biomarker in metastatic hormone receptor-positive breast cancer. Breast Cancer Res. (2021) 23:85. doi: 10.1186/s13058-021-01462-3 34392831 PMC8365900

[6] Nunnery SE Mayer IA . Targeting the PI3K/AKT/mTOR pathway in hormone-positive breast cancer. Drugs. (2020) 80:1685–97. doi: 10.1007/s40265-020-01394-w 32894420 PMC7572750

[7] Skolariki A D’Costa J Little M Lord S . Role of PI3K/Akt/mTOR pathway in mediating endocrine resistance: concept to clinic. Explor Targeted Anti-Tumor Ther. (2022) 3:172. doi: 10.37349/etat.2022.00078 36046843 PMC9400772

[8] Palafox M Monserrat L Bellet M Villacampa G Gonzalez-Perez A Oliveira M . High p16 expression and heterozygous RB1 loss are biomarkers for CDK4/6 inhibitor resistance in ER+ breast cancer. Nat Commun. (2022) 13:5258. doi: 10.1038/s41467-022-32828-6 36071033 PMC9452562

[9] Brechbuhl HM Han A Paul KV Nemkov T Ramachandran S Ward A . Metabolic switch in endocrine resistant estrogen receptor positive breast cancer. bioRxiv. (2024). doi: 10.1101/2024.12.28.630631 39763830 PMC11703175

[10] Ward AV Riley D Cosper KE Finlay-Schultz J Brechbuhl HM Libby AE . Lipid metabolic reprogramming drives triglyceride storage and variable sensitivity to FASN inhibition in endocrine-resistant breast cancer cells. Breast Cancer Res. (2025) 27:32. doi: 10.1186/s13058-025-01991-1 40055794 PMC11889759

[11] Ahn S Park JH Grimm SL Piyarathna DWB Samanta T Putluri V . Metabolomic rewiring promotes endocrine therapy resistance in breast cancer. Cancer Res. (2024) 84:291–304. doi: 10.1158/0008-5472.CAN-23-0184 37906431 PMC10842725

[12] Kim N Lukong KE . Treating ER-positive breast cancer: a review of the current FDA-approved SERMs and SERDs and their mechanisms of action. Oncol Rev. (2025) 19:1564642. doi: 10.3389/or.2025.1564642 40275985 PMC12018393

[13] Xu J Iwabuchi E Miki Y Kanai A Takagi K Suzuki T . FE65 defines the efficacy of tamoxifen treatment via osteopontin expression in estrogen receptor-positive breast cancer. Pathol Res Pract. (2022) 234:153898. doi: 10.1016/j.prp.2022.153898 35447603

[14] Downton T Zhou F Segara D Jeselsohn R Lim E . Oral selective estrogen receptor degraders (SERDs) in breast cancer: advances, challenges, and current status. Drug Des Dev Ther. (2022), 2933–48. doi: 10.2147/DDDT.S380925 36081610 PMC9447452

[15] Zhang G Ni H Mishieva L Bartels S Christgen M Christgen H . Integrative analysis of RNA expression signatures and recurrent genomic alterations before treatment: link to menopausal status, short-term endocrine therapy response and disease-free survival in luminal breast cancer. ESMO Open. (2025) 10:105913. doi: 10.1016/j.esmoop.2025.105913 41274166 PMC12681821

[16] González-Ruíz J Terry MB Cabrera-Galeana P Monroy-Chargoy A Horowitz C Bickel N . Predictors of endocrine resistance in a cohort of Mexican breast cancer patients. Res Square. (2024):rs-3. doi: 10.21203/rs.3.rs-4414887/v1 38853915 PMC11160913

[17] Hartkopf AD Grischke EM Brucker SY . Endocrine-resistant breast cancer: mechanisms and treatment. Breast Care. (2020) 15:347–54. doi: 10.1159/000508675 32982644 PMC7490658

[18] Cvetanović AS Jankovic KB Stojković AS Živković ND Kostić MS Popović LS . Real-world clinical experience of first-line ribociclib combined with an aromatase inhibitor in metastatic breast cancer. Cancers. (2026) 18:242. doi: 10.3390/cancers18020242 41595162 PMC12838554

[19] Kim SS Lee MH Lee MO . Histone methyltransferases regulate the transcriptional expression of ERα and the proliferation of tamoxifen-resistant breast cancer cells. Breast Cancer Res Treat. (2020) 180:45–54. doi: 10.1007/s10549-019-05517-0 31897900 PMC7031178

[20] Zhang X Yang F Huang Z Liu X Xia G Huang J . Macrophages promote subtype conversion and endocrine resistance in breast cancer. Cancers. (2024) 16:678. doi: 10.3390/cancers16030678 38339428 PMC10854660

[21] Liu L Jiang D Bai S Zhang X Kang Y . Research progress of exosomes in drug resistance of breast cancer. Front Bioeng Biotechnol. (2024) 11:1214648. doi: 10.3389/fbioe.2023.1214648 38239920 PMC10794616

[22] Goto W Kashiwagi S Iimori N Kouhashi R Yabumoto A Takada K . Eribulin treatment promotes re-expression of estrogen receptor in endocrine therapy-resistant hormone receptor-positive breast cancer cells. Anticancer Res. (2023) 43:603–11. doi: 10.21873/anticanres.16196 36697070

[23] Hu K Wu Y Huang Y Zhou M Wang Y Huang X . Annotation-free deep learning algorithm trained on hematoxylin & eosin images predicts epithelial-to-mesenchymal transition phenotype and endocrine response in estrogen receptor-positive breast cancer. Breast Cancer Res. (2025) 27:6. doi: 10.1186/s13058-025-01959-1 39800743 PMC11725188

[24] El-Sehrawy AAMA Hsu CY Alkhathami AG Chandra M Basunduwah TS Malathi H . (2025). Metabolic reprogramming: The driving force behind cancer drug resistance, in: Seminars in Oncology ( WB Saunders), 52 (5):152392. doi: 10.1016/j.seminoncol.2025.152392 40674808

[25] Bao MHR Wong CCL . Hypoxia, metabolic reprogramming, and drug resistance in liver cancer. Cells. (2021) 10:1715. doi: 10.3390/cells10071715 34359884 PMC8304710

[26] Lv L Yang S Zhu Y Zhai X Li S Tao X . Relationship between metabolic reprogramming and drug resistance in breast cancer. Front Oncol. (2022) 12:942064. doi: 10.3389/fonc.2022.942064 36059650 PMC9434120

[27] Polónia B Xavier CP Kopecka J Riganti C Vasconcelos MH . The role of extracellular vesicles in glycolytic and lipid metabolic reprogramming of cancer cells: consequences for drug resistance. Cytokine Growth Factor Rev. (2023) 73:150–62. doi: 10.1016/j.cytogfr.2023.05.001 37225643

[28] Yang R Yi M Xiang B . Novel insights on lipid metabolism alterations in drug resistance in cancer. Front Cell Dev Biol. (2022) 10:875318. doi: 10.3389/fcell.2022.875318 35646898 PMC9136290

[29] Fu W Sun A Dai H . Lipid metabolism involved in progression and drug resistance of breast cancer. Genes Dis. (2025) 12:101376. doi: 10.1016/j.gendis.2024.101376 40256431 PMC12008617

[30] Li X Guo R Yang G Zhu L Liu R Xia L . Research progress of lipid metabolism reprogramming and related drug therapy in ovarian cancer. J Steroid Biochem Mol Biol. (2025) 253:106816. doi: 10.1016/j.jsbmb.2025.106816 40532958

[31] Wu Y Pi D Zhou S Yi Z Dong Y Wang W . Ginsenoside Rh3 induces pyroptosis and ferroptosis through the Stat3/p53/NRF2 axis in colorectal cancer cells: ginsenoside Rh3 has anti-colorectal cancer properties. Acta Biochim Biophys Sin. (2023) 55:587. doi: 10.3724/abbs.2023068 37092860 PMC10195137

[32] Li R Li Y . Role of metabolic reprogramming of CAFs in tumor development and progression. Int J Oncol. (2025) 67:90. doi: 10.3892/ijo.2025.5796 40878935 PMC12425346

[33] Zhang L Zhao J Su C Wu J Jiang L Chi H . Organoid models of ovarian cancer: resolving immune mechanisms of metabolic reprogramming and drug resistance. Front Immunol. (2025) 16:1573686. doi: 10.3389/fimmu.2025.1573686 40191206 PMC11968360

[34] Shi J Shen Y Zhang J . Emerging roles of small extracellular vesicles in metabolic reprogramming and drug resistance in cancers. Cancer Drug Resistance. (2024) 7:38. doi: 10.20517/cdr.2024.81 39403606 PMC11472704

[35] Zhu Y Yan W Tong L Yang J Ge S Fan J . Metabolic reprogramming: a crucial contributor to anticancer drug resistance. MedComm. (2025) 6:e70358. doi: 10.1002/mco2.70358 40919131 PMC12413565

[36] Liu S Zhang X Wang W Li X Sun X Zhao Y . Metabolic reprogramming and therapeutic resistance in primary and metastatic breast cancer. Mol Cancer. (2024) 23:261. doi: 10.1186/s12943-024-02165-x 39574178 PMC11580516

[37] Kumari S Srilatha M Nagaraju GP . Understanding the role of the microbiome in breast cancer progression. Crit Rev Oncog. (2025) 30(2). doi: 10.1615/CritRevOncog.2024056468 40561428

[38] Wu X Tan X Bao Y Yan W Zhang Y . Landscape of metabolic alterations and treatment strategies in breast cancer. Genes Dis. (2025) 12:101521. doi: 10.1016/j.gendis.2025.101521 40548063 PMC12179608

[39] Noh SW Ryu HS Kim YH Oh BC . SGLT2 inhibitors as systemic metabolic modulators: linking glucose excretion to liver function restoration. Endocrinol Metab. (2025) 40:851–65. doi: 10.3803/EnM.2025.2786 41479267 PMC12765894

[40] Li J Huang Y Chang R Ni A Fang L Zhou X . Developmental and metabolic toxicity of diphenyl phosphate: insights from an integrative mechanistic framework in zebrafish embryos. Environ pollut. (2026), 127672. doi: 10.1016/j.envpol.2026.127672 41534654

[41] Ocaña MC Martínez-Poveda B Quesada AR Medina MÁ . Glucose favors lipid anabolic metabolism in the invasive breast cancer cell line MDA-MB-231. Biology. (2020) 9:16. doi: 10.3390/biology9010016 31936882 PMC7168317

[42] Hu JJ Zhang QY Yang ZC . The correlation between obesity and the occurrence and development of breast cancer. Eur J Med Res. (2025) 30:419. doi: 10.1186/s40001-025-02659-4 40414892 PMC12105307

[43] Menendez JA Mehmi I Papadimitropoulou A Vander Steen T Cuyàs E Verdura S . Fatty acid synthase is a key enabler for endocrine resistance in heregulin-overexpressing luminal b-like breast cancer. Int J Mol Sci. (2020) 21:7661. doi: 10.3390/ijms21207661 33081219 PMC7588883

[44] Zhang J Song Y Shi Q Fu L . Research progress on FASN and MGLL in the regulation of abnormal lipid metabolism and the relationship between tumor invasion and metastasis. Front Med. (2021) 15:649–56. doi: 10.1007/s11684-021-0830-0 33973101

[45] Naz T Zhao XY Li S Saeed T Ullah S Nazir Y . The interplay of transcriptional regulator SREBP1 with AMPK promotes lipid biosynthesis in Mucor circinelloides WJ11. Biochim Biophys Acta (BBA)-Molecular Cell Biol Lipids. (2025) 1870:159592. doi: 10.1016/j.bbalip.2024.159592 39733936

[46] Singhai H Rathee S Patil UK . Lipidomics in breast cancer: decoding metabolic reprogramming and unlocking therapeutic opportunities. Curr Drug Targets. (2025) 26(11):770–8. doi: 10.2174/0113894501387287250611095023 40600551

[47] Nishimoto K Okahashi N Maruyama M Izumi Y Nakatani K Ito Y . Lipidome and metabolome analyses reveal metabolic alterations associated with MCF-7 apoptosis upon 4-hydroxytamoxifen treatment. Sci Rep. (2023) 13:18549. doi: 10.1038/s41598-023-45764-2 37899460 PMC10613619

[48] Fu Y Zou T Shen X Nelson PJ Li J Wu C . Lipid metabolism in cancer progression and therapeutic strategies. MedComm. (2021) 2:27–59. doi: 10.1002/mco2.27 34766135 PMC8491217

[49] Chen X Chen S Yu D . Metabolic reprogramming of chemoresistant cancer cells and the potential significance of metabolic regulation in the reversal of cancer chemoresistance. Metabolites. (2020) 10:289. doi: 10.3390/metabo10070289 32708822 PMC7408410

[50] Wang C Chen Z Yi Y Ding Y Xu F Kang H . RBM45 reprograms lipid metabolism promoting hepatocellular carcinoma via Rictor and ACSL1/ACSL4. Oncogene. (2024) 43:328–40. doi: 10.1038/s41388-023-02902-4 38040804

[51] Simeone P Tacconi S Longo S Lanuti P Bravaccini S Pirini F . Expanding roles of de novo lipogenesis in breast cancer. Int J Environ Res Public Health. (2021) 18:3575. doi: 10.3390/ijerph18073575 33808259 PMC8036647

[52] Ferraro GB Ali A Luengo A Kodack DP Deik A Abbott KL . Fatty acid synthesis is required for breast cancer brain metastasis. Nat Cancer. (2021) 2:414–28. doi: 10.1038/s43018-021-00183-y 34179825 PMC8223728

[53] Tiwari U Akhtar S Mir SS Khan MKA . SREBP-1 in obesity-induced breast cancer: mechanisms and therapeutic perspectives. Mol Biol Rep. (2025) 52:663. doi: 10.1007/s11033-025-10775-x 40593359

[54] Hou YJ Yang XX Meng HX . Mitochondrial metabolism in laryngeal cancer: therapeutic mechanisms and prospects. Biochim Biophys Acta (BBA)-Reviews Cancer. (2025) 1880:189335. doi: 10.1016/j.bbcan.2025.189335 40311711

[55] Wang H Yun Z Cao Y Li X Liu Z Pai CP . PML1-mediated feedforward loop through PI3K and MAPK axes drives endocrine resistance. bioRxiv. (2025) 2025-05. doi: 10.1101/2025.05.15.653365 40463196 PMC12132319

[56] Li X Zhang Y Zhang T Zhao L Lin CG Hu H . Tafazzin mediates tamoxifen resistance by regulating cellular phospholipid composition in ER-positive breast cancer. Cancer Gene Ther. (2024) 31:69–81. doi: 10.1038/s41417-023-00683-2 37935981

[57] Zhang M Junchen PAN Huang P . Interaction between RAS gene and lipid metabolism in cancer. J Zhejiang Univ (Medical Sciences). (2021) 50:17. doi: 10.3724/zdxbyxb-2021-0054 34117852 PMC8675076

[58] Asperger H Stamm N Gierke B Pawlak M Hofmann U Zanger UM . Progesterone receptor membrane component 1 regulates lipid homeostasis and drives oncogenic signaling resulting in breast cancer progression. Breast Cancer Res. (2020) 22:75. doi: 10.1186/s13058-020-01312-8 32660617 PMC7359014

[59] Yang Y Zhang E Mao X Liu G Pan Y . Targeting palmitoylation: A novel frontier in cancer biology and immunotherapy. Biochim Biophys Acta (BBA)-Reviews Cancer. (2025), 189509. doi: 10.1016/j.bbcan.2025.189509 41365469

[60] Closset L Foy JP Louadj L Pramil E Marangoni E Bieche I . CD36 enhances sensitivity of triple negative breast cancer cells to palmitate-induced ferroptosis. Cell Death Dis. (2026) 17(1):219. doi: 10.1038/s41419-026-08460-3 41672971 PMC12920903

[61] Pakiet A Ciosek M Lange O Duzowska K Janczy A Kapusta M . Very long-chain fatty acids accumulate in breast cancer tissue and serum. Cancer Cell Int. (2025) 25:296. doi: 10.1186/s12935-025-03928-2 40759952 PMC12320370

[62] Terry AR Nogueira V Rho H Ramakrishnan G Li J Kang S . CD36 maintains lipid homeostasis via selective uptake of monounsaturated fatty acids during matrix detachment and tumor progression. Cell Metab. (2023) 35:2060–76. doi: 10.1016/j.cmet.2023.09.012 37852255 PMC11748917

[63] Wu L Ou GL Zhang W Ma HX Li XY Zhen YH . Fatty acid-binding proteins in cancers. Int J Surg. (2025) 111:8402–22. doi: 10.1097/JS9.0000000000003049 40717587 PMC12626542

[64] Smolińska K Szopa A Dobrowolski P Dziedzic J Nowak N Serefko A . Targeting fatty acid-binding protein 4: A therapeutic intersection of obesity and cancer via lipid metabolism. Prog Lipid Res. (2025), 101353. doi: 10.1016/j.plipres.2025.101353 40934976

[65] Warren WG Osborn M Yates A Wright K O'Sullivan SE . The emerging role of fatty acid binding protein 5 (FABP5) in cancers. Drug Discov Today. (2023) 28:103628. doi: 10.1016/j.drudis.2025.104504 37230284

[66] Jia J Bai C Zhang W Xu J Hu T . Highly expressed fatty acid‐binding protein 6 mediates lipid metabolism remodeling in tumor cells via intracellular bile acid transport to promote pancreatic cancer metastasis. Mol Carcinog. (2026) 65(3):343–55. doi: 10.1002/mc.70076 41496320

[67] Borús DL Zadra G Minsky D Costa ML Córsico B Storch J . Fatty acid binding protein 1 (FABP1) depletion promotes an oxidative metabolic shift in Caco-2 colorectal cancer cells. Biochim Biophys Acta (BBA)-Molecular Cell Biol Lipids. (2025) 1870:159661. doi: 10.1016/j.bbalip.2025.159661 40639772 PMC12361919

[68] Surendran A Jamalkhah M Poutou J Birtch R Lawson C Dave J . Fatty acid transport protein inhibition sensitizes breast and ovarian cancers to oncolytic virus therapy via lipid modulation of the tumor microenvironment. Front Immunol. (2023) 14:1099459. doi: 10.3389/fimmu.2023.1099459 36969187 PMC10036842

[69] Wang C Han J Chen Y . Inhibition of CD36 and Nogo-B expression inhibited the proliferation and migration of triple negative breast cancer cells. Sheng Wu Gong Cheng Xue Bao = Chin J Biotechnol. (2023) 39:4168–88. doi: 10.13345/j.cjb.230050 37877398

[70] He M Xu S Yan F Ruan J Zhang X . Fatty acid metabolism: a new perspective in breast cancer precision therapy. Front Bioscience-Landmark. (2023) 28:348. doi: 10.31083/j.fbl2812348 38179746

[71] Mukherjee A Bilecz AJ Lengyel E . The adipocyte microenvironment and cancer. Cancer Metastasis Rev. (2022) 41:575–87. doi: 10.1007/s10555-022-10059-x 35941408

[72] Li F Huang H Huang Q Mao X Cong N Zhuang W . Necrotic and apoptotic adipocytes in the hypoxic tumor microenvironment supply triglycerides to induce cisplatin resistance in the metastatic lymph nodes of head and neck carcinoma. Cell Death Dis. (2025) 16:854. doi: 10.1038/s41419-025-08239-y 41285714 PMC12644729

[73] Zhou X Zhang J Lv W Zhao C Xia Y Wu Y . The pleiotropic roles of adipocyte secretome in remodeling breast cancer. J Exp Clin Cancer Res. (2022) 41:203. doi: 10.1186/s13046-022-02408-z 35701840 PMC9199207

[74] Wang J Mi S Ding M Li X Yuan S . Metabolism and polarization regulation of macrophages in the tumor microenvironment. Cancer Lett. (2022) 543:215766. doi: 10.1016/j.canlet.2022.215766 35690285

[75] Lee-Rueckert M Jauhiainen M Kovanen PT Escolà-Gil JC . Lipids and lipoproteins in the interstitial tissue fluid regulate the formation of dysfunctional tissue-resident macrophages: Implications for atherogenic, tumorigenic, and obesogenic processes. In: Seminars in Cancer Biology, Academic Press (2025) 114:104–27. doi: 10.1016/j.semcancer.2025.06.008 40545184

[76] Tian J Zhang L La X Fan X Li A Wu C . Tumor-secreted GRP78 induces M2 polarization of macrophages by promoting lipid catabolism. Cell Signalling. (2023) 108:110719. doi: 10.1016/j.cellsig.2023.110719 37207940

[77] Liu MK Cheng LL Yi HM He Y Li X Fu D . Enhanced lipid metabolism confers the immunosuppressive tumor microenvironment in CD5-positive non-MYC/BCL2 double expressor lymphoma. Front Oncol. (2022) 12:885011. doi: 10.3389/fonc.2022.885011 36276140 PMC9583025

[78] Jonker PB Muir A . Metabolic ripple effects–deciphering how lipid metabolism in cancer interfaces with the tumor microenvironment. Dis Models Mech. (2024) 17:dmm050814. doi: 10.1242/dmm.050814 39284708 PMC11423921

[79] Ishida CT Myers SL Kubota CS Shao W McGuire MR Liu C . SREBP-dependent regulation of lipid homeostasis is required for progression and growth of pancreatic ductal adenocarcinoma. Cancer Res Commun. (2024) 4:2539–52. doi: 10.1101/2024.02.04.578802 39240063 PMC11444119

[80] Yang R Liu S Bi Y Jin R Ye Z Cai X . The role of lipid metabolic interactions in reshaping the tumor microenvironment. Acta Biochim Biophys Sin. (2025) 58(4):725. doi: 10.3724/abbs.2025166 41098086 PMC13107023

[81] Dai CL Qiu ZY Wang AQ Yan S Zhang LJ Luan X . Targeting cholesterol metabolism: a promising therapy strategy for cancer. Acta Pharmacol Sin. (2025) 46:2093–104. doi: 10.1038/s41401-025-01531-9 40133625 PMC12274607

[82] Ning S Liu C Wang K Cai Y Ning Z Li M . NUCB2/Nesfatin-1 drives breast cancer metastasis through the up-regulation of cholesterol synthesis via the mTORC1 pathway. J Transl Med. (2023) 21:362. doi: 10.1186/s12967-023-04236-x 37277807 PMC10243030

[83] Xu M Hu J Pu L Liu J Yang Y Li Q . Multi-omics integration identifies the cholesterol metabolic enzyme DHCR24 as a key driver in breast cancer. Biology. (2025) 15:40. doi: 10.3390/biology15010040 41514881 PMC12785013

[84] Yang Y Gao T Yuan B Hao X Huo M Hu T . KDM5B cooperates with CRL4B complex to promote the tumorigenesis of ER+ breast cancer via regulating cholesterol metabolism. Cell Death Dis. (2026) 17:207. doi: 10.1038/s41419-026-08438-1 41654562 PMC12894857

[85] Ghanbari F Fortier AM Park M Philip A . Cholesterol-induced metabolic reprogramming in breast cancer cells is mediated via the ERRα pathway. Cancers. (2021) 13:2605. doi: 10.3390/cancers13112605 34073320 PMC8198778

[86] Antipenko ID Makarova JA Shkurnikov MY Tonevitsky AG . The impact of IGFBP6 knockdown on cholesterol metabolism in breast cancer cells. Curr Med Chem. (2025) 33(15):2969–83. doi: 10.2174/0109298673396050250729132235 40947699

[87] Feldt M Menard J Rosendahl AH Lettiero B Bendahl PO Belting M . The effect of statin treatment on intratumoral cholesterol levels and LDL receptor expression: a window-of-opportunity breast cancer trial. Cancer Metab. (2020) 8:25. doi: 10.1186/s40170-020-00231-8 33292612 PMC7682108

[88] Tuokkola JE Reese LE Wang Y O’Connor CH VanTreeck JG Rumahorbo AH . Fibroblast growth factor receptor signaling modulates cholesterol storage in a SOAT1-dependent manner to promote mammary tumor cell invasion. Breast Cancer Res. (2025) 27:132. doi: 10.1186/s13058-025-02084-9 40665359 PMC12261779

[89] González-Ortiz A Galindo-Hernández O Hernández-Acevedo GN Hurtado-Ureta G García-González V . Impact of cholesterol-pathways on breast cancer development, a metabolic landscape. J Cancer. (2021) 12:4307. doi: 10.7150/jca.54637 34093831 PMC8176427

[90] Ghanbari F Mader S Philip A . Cholesterol as an endogenous ligand of ERRα promotes ERRα-mediated cellular proliferation and metabolic target gene expression in breast cancer cells. Cells. (2020) 9:1765. doi: 10.3390/cells9081765 32717915 PMC7463712

[91] Luo M Bao L Chen Y Xue Y Wang Y Zhang B . ZMYND8 is a master regulator of 27-hydroxycholesterol that promotes tumorigenicity of breast cancer stem cells. Sci Adv. (2022) 8:eabn5295. doi: 10.1126/sciadv.abn5295 35857506 PMC9286501

[92] Yaduvanshi H Deshmukh B Singh A Mayengbam SS Bhat MK . Anti-tumor response by targeting glucose metabolism and depletion of membrane cholesterol in breast cancer and melanoma cells. Biochim Biophys Acta (BBA)-Molecular Cell Res. (2025) 120088. doi: 10.1016/j.bbamcr.2025.120088 41253196

[93] Urano Y Noguchi N . Enzymatically formed oxysterols and cell death. In: Implication of Oxysterols and Phytosterols in Aging and Human Diseases (2023) 193–211. doi: 10.1007/978-3-031-43883-7_10 38036881

[94] Moldavski O Zushin PJH Berdan CA Van Eijkeren RJ Jiang X Qian M . 4β-Hydroxycholesterol is a prolipogenic factor that promotes SREBP1c expression and activity through the liver X receptor. J Lipid Res. (2021) 62:100051. doi: 10.1016/j.jlr.2021.100051 33631213 PMC8042401

[95] George M Lang M Gali CC Babalola JA Tam-Amersdorfer C Stracke A . Liver X receptor activation attenuates oxysterol-induced inflammatory responses in fetoplacental endothelial cells. Cells. (2023) 12:1186. doi: 10.3390/cells12081186 37190095 PMC10137015

[96] Griffiths WJ Wang Y . Sterols, oxysterols, and accessible cholesterol: signalling for homeostasis, in immunity and during development. Front Physiol. (2021) 12:723224. doi: 10.3389/fphys.2021.723224 34690800 PMC8531217

[97] Kloudova‐Spalenkova A Holy P Soucek P . Oxysterols in cancer management: From therapy to biomarkers. Br J Pharmacol. (2021) 178:3235–47. doi: 10.1111/bph.15273 32986851

[98] Holý P Brynychová V Šeborová K Haničinec V Koževnikovová R Trnková M . Integrative analysis of mRNA and miRNA expression profiles and somatic variants in oxysterol signaling in early‐stage luminal breast cancer. Mol Oncol. (2023) 17:2074–89. doi: 10.1002/1878-0261.13495 37491786 PMC10552891

[99] Molostvov G Gachechiladze M Shaaban AM Hayward S Dean I Dias IH . Tspan6 stimulates the chemoattractive potential of breast cancer cells for B cells in an EV-and LXR-dependent manner. Cell Rep. (2023) 42(3). doi: 10.1016/j.celrep.2023.112207 36867531

[100] Zhang Y Chen Y Zhuang C Qi J Zhao RC Wang J . Lipid droplets in the nervous system: involvement in cell metabolic homeostasis. Neural Regener Res. (2025) 20:740–50. doi: 10.4103/NRR.NRR-D-23-01401 38886939 PMC11433920

[101] Holzhütter HG . Dynamical modelling of lipid droplet formation suggests a key function of membrane phospholipids. FEBS J. (2025) 292:206–25. doi: 10.1111/febs.17238 39132700 PMC11705222

[102] Cabodevilla AG Son N Goldberg IJ . Intracellular lipase and regulation of the lipid droplet. Curr Opin Lipidology. (2024) 35:85–92. doi: 10.1097/MOL.0000000000000918 38447014 PMC10919935

[103] Roberts MA Olzmann JA . Protein quality control and lipid droplet metabolism. Annu Rev Cell Dev Biol. (2020) 36:115–39. doi: 10.1146/annurev-cellbio-031320-101827 33021827 PMC7593838

[104] Doncheva AI Li Y Khanal P Hjorth M Kolset SO Norheim FA . Altered hepatic lipid droplet morphology and lipid metabolism in fasted Plin2-null mice. J Lipid Res. (2023) 64:100461. doi: 10.1016/j.jlr.2023.100461 37844775 PMC10716011

[105] Zhou L Song Z Hu J Liu L Hou Y Zhang X . ACSS3 represses prostate cancer progression through downregulating lipid droplet-associated protein PLIN3. Theranostics. (2021) 11:841. doi: 10.7150/thno.49384 33391508 PMC7738848

[106] Ischebeck T Krawczyk HE Mullen RT Dyer JM Chapman KD . Lipid droplets in plants and algae: distribution, formation, turnover and function. In: Seminars in Cell & Developmental Biology, (2020) 108:82–93. doi: 10.1016/j.semcdb.2020.02.014 32147380

[107] Xu Y Zhang Y Sun W Tang Q Feng W Xiao H . Characteristics of different lipid droplet-mitochondrial contacts patterns during lipid droplet metabolism in T2DM-induced MASLD. Sci Rep. (2025) 15:3399. doi: 10.1038/s41598-025-87871-2 39870911 PMC11772659

[108] Bacci M Lorito N Smiriglia A Subbiani A Bonechi F Comito G . Acetyl-CoA carboxylase 1 controls a lipid droplet–peroxisome axis and is a vulnerability of endocrine-resistant ER+ breast cancer. Sci Transl Med. (2024) 16:eadf9874. doi: 10.1126/scitranslmed.adf9874 38416843

[109] Ralhan I Chang CL Lippincott-Schwartz J Ioannou MS . Lipid droplets in the nervous system. J Cell Biol. (2021) 220:e202102136. doi: 10.1083/jcb.202102136 34152362 PMC8222944

[110] Diep DTV Bohnert M . The vacuole lipid droplet contact site vCLIP. Contact. (2024) 7:25152564241308722. doi: 10.1177/25152564241308722 39717764 PMC11664512

[111] Andolino C Cotul EK Xianyu Z Li Y Bhat D Ayers M . Fatty acid synthase-derived lipid stores support breast cancer metastasis. Cancer Metab. (2025) 13:35. doi: 10.21203/rs.3.rs-5510550/v1 40640944 PMC12247306

[112] Rossi T Zamponi R Chirico M Pisanu ME Iorio E Torricelli F . BETi enhance ATGL expression and its lipase activity to exert their antitumoral effects in triple-negative breast cancer (TNBC) cells. J Exp Clin Cancer Res. (2023) 42:7. doi: 10.1186/s13046-022-02571-3 36604676 PMC9817244

[113] Meng Y Guo D Lin L Zhao H Xu W Luo S . Glycolytic enzyme PFKL governs lipolysis by promoting lipid droplet–mitochondria tethering to enhance β-oxidation and tumor cell proliferation. Nat Metab. (2024) 6:1092–107. doi: 10.1038/s42255-024-01047-2 38773347

[114] Zhu R Yang Y Shao F Wang J Gao Y He J . Choline kinase alpha2 promotes lipid droplet lipolysis in non-small-cell lung carcinoma. Front Oncol. (2022) 12:848483. doi: 10.3389/fonc.2022.848483 35463311 PMC9021865

[115] Jusović M Starič P Jarc Jovičić E Petan T . The combined inhibition of autophagy and diacylglycerol acyltransferase-mediated lipid droplet biogenesis induces cancer cell death during acute amino acid starvation. Cancers. (2023) 15:4857. doi: 10.3390/cancers15194857 37835551 PMC10571868

[116] Obaseki E Adebayo D Bandyopadhyay S Hariri H . Lipid droplets and fatty acid‐induced lipotoxicity: in a nutshell. FEBS Lett. (2024) 598:1207–14. doi: 10.1002/1873-3468.14808 38281809 PMC11126361

[117] Prevost CT Gansereit WB Kashatus DF . NRF2 regulates lipid droplet dynamics to prevent lipotoxicity. iScience. (2025) 28(7). doi: 10.1016/j.isci.2025.112925 40678507 PMC12270665

[118] Cortini M Ilieva E Massari S Bettini G Avnet S Baldini N . Uncovering the protective role of lipid droplet accumulation against acid-induced oxidative stress and cell death in osteosarcoma. Biochim Biophys Acta (BBA)-Molecular Basis Dis. (2025) 1871:167576. doi: 10.1016/j.bbadis.2024.167576 39561857

[119] Bång-Rudenstam A Cerezo-Magaña M Horvath M Talbot H Gustafsson E Jonathan S . Tumour acidosis remodels the glycocalyx to control lipid scavenging and ferroptosis. Nat Cell Biol. (2026) 28(3):567–80. doi: 10.1038/s41556-026-01879-y 41673170 PMC12992114

[120] Kostecka LG Mendez S Li M Khare P Zhang C Le A . Cancer cells employ lipid droplets to survive toxic stress. Prostate. (2024) 84:644–55. doi: 10.1002/pros.24680 38409853

[121] Almanza A Mnich K Blomme A Robinson CM Rodriguez-Blanco G Kierszniowska S . Regulated IRE1α-dependent decay (RIDD)-mediated reprograming of lipid metabolism in cancer. Nat Commun. (2022) 13:2493. doi: 10.1038/s41467-022-30159-0 35524156 PMC9076827

[122] Petan T . Lipid droplets in cancer. In: Organelles in Disease (2020) 53–86. doi: 10.1007/112_2020_51 33074407

[123] Pillai S Mahmud I Mahar R Griffith C Langsen M Nguyen J . Lipogenesis mediated by OGR1 regulates metabolic adaptation to acid stress in cancer cells via autophagy. Cell Rep. (2022) 39(6). doi: 10.1016/j.celrep.2022.110796 35545051 PMC9137419

[124] Li X Yu X Huang K Yu X Luo S Huang X . TM9SF1 drives the lipophagic flux via AMPK-ULK1 signaling to sustain metabolic fitness in HER2-positive breast cancer. Cell Death Dis. (2025) 16:755. doi: 10.1038/s41419-025-08093-y 41136362 PMC12552452

[125] Labbé K LeBon L King B Vu N Stoops EH Ly N . Specific activation of the integrated stress response uncovers regulation of central carbon metabolism and lipid droplet biogenesis. Nat Commun. (2024) 15:8301. doi: 10.1038/s41467-024-52538-5 39333061 PMC11436933

[126] Liu X Sun X Mu W Li Y Bu W Yang T . Autophagic flux-lipid droplet biogenesis cascade sustains mitochondrial fitness in colorectal cancer cells adapted to acidosis. Cell Death Discov. (2025) 11:21. doi: 10.1038/s41420-025-02301-6 39856069 PMC11761495

[127] Lasheras-Otero I Feliu I Maillo A Moreno H Redondo-Munoz M Aldaz P . The regulators of peroxisomal acyl-carnitine shuttle CROT and CRAT promote metastasis in melanoma. J Invest Dermatol. (2023) 143:305–16. doi: 10.1016/j.jid.2022.08.038 36058299

[128] Sanclemente-Cardoza V Estela-Zape JL . Mitochondrial adaptations in acute respiratory distress syndrome. Rev Médica del Instituto Mexicano del Seguro Soc. (2024) 62:e5450. doi: 10.5281/zenodo.10998887 39528425 PMC12240607

[129] Kolb H Kempf K Röhling M Lenzen-Schulte M Schloot NC Martin S . Ketone bodies: from enemy to friend and guardian angel. BMC Med. (2021) 19:313. doi: 10.1186/s12916-021-02185-0 34879839 PMC8656040

[130] Cardoso AC Lam NT Savla JJ Nakada Y Pereira AHM Elnwasany A . Mitochondrial substrate utilization regulates cardiomyocyte cell-cycle progression. Nat Metab. (2020) 2:167–78. doi: 10.1038/s42255-020-0169-x 32617517 PMC7331943

[131] Wang Y Sun W Yan S Meng Z Jia M Tian S . A new strategy to alleviate the obesity induced by endocrine disruptors—a unique lysine metabolic pathway of nanoselenium Siraitia grosvenorii to repair gut microbiota and resist obesity. Food Chem Toxicol. (2023) 175:113737. doi: 10.1016/j.fct.2023.113737 36944396

[132] Rashed N Liu W Luo X . The role of fatty acid oxidation in the tumor microenvironment: Implications for cancer progression and therapeutic strategies. Biochim Biophys Acta (BBA)-Reviews Cancer. (2025), 189474. doi: 10.1016/j.bbcan.2025.189474 41075849

[133] Luo E Agarwal NM Choi J . Metabolomic profiling reveals key metabolic alterations in MCF7 tamoxifen-resistant cells following EPAS1 inhibition. J Proteome Res. (2025) 24:4070–81. doi: 10.1021/acs.jproteome.5c00170 40675783

[134] Wang C Uray IP Mazumdar A Mayer JA Brown PH . SLC22A5/OCTN2 expression in breast cancer is induced by estrogen via a novel intronic estrogen-response element (ERE). Breast Cancer Res Treat. (2012) 134:101–15. doi: 10.1007/s10549-011-1925-0 22212555 PMC3416040

[135] Wang J Scholtens D Holko M Ivancic D Lee O Hu H . Lipid metabolism genes in contralateral unaffected breast and estrogen receptor status of breast cancer. Cancer Prev Res. (2013) 6:321–30. doi: 10.1158/1940-6207.CAPR-12-0304 23512947

[136] Tawbeh A Gondcaille C Saih FE Raas Q Loichot D Hamon Y . Impaired peroxisomal beta-oxidation in microglia triggers oxidative stress and impacts neurons and oligodendrocytes. Front Mol Neurosci. (2025) 18:1542938. doi: 10.3389/fnmol.2025.1542938 39958993 PMC11826809

[137] Houten SM Denis S Argmann CA Jia Y Ferdinandusse S Reddy JK . Peroxisomal L-bifunctional enzyme (Ehhadh) is essential for the production of medium-chain dicarboxylic acids. J Lipid Res. (2012) 53:1296–303. doi: 10.1194/jlr.m024463 22534643 PMC3371241

[138] Al-Shami K Awadi S Khamees AA Alsheikh AM Al-Sharif S Ala’Bereshy R . Estrogens and the risk of breast cancer: A narrative review of literature. Heliyon. (2023) 9(9). doi: 10.1016/j.heliyon.2023.e20224 37809638 PMC10559995

[139] Weryha G Pascal-Vigneron V Klein M Leclère J . Selective estrogen receptor modulators. Curr Opin Rheumatol. (1999) 11:301–6. doi: 10.1097/00002281-199907000-00014 10411386

[140] Wang J Shidfar A Ivancic D Ranjan M Liu L Choi MR . Overexpression of lipid metabolism genes and PBX1 in the contralateral breasts of women with estrogen receptor‐negative breast cancer. Int J Cancer. (2017) 140:2484–97. doi: 10.1002/ijc.30680 28263391

[141] Zhang F Chen Y Long J Dong L Wang Y . Effect of n-3 and n-6 polyunsaturated fatty acids on lipid metabolic genes and estrogen receptor expression in MCF-7 breast cancer cells. Clin Lab. (2015) 61:397–403. doi: 10.7754/clin.lab.2014.140902 25975008

[142] Bonechi F Bacci M Lorito N Smiriglia A Subbiani A Pagliantini E . ESR1-activating mutations confer metabolic vulnerabilities in ER+ breast cancer. Cancer Res. (2026) 86:679–92. doi: 10.1158/0008-5472.CAN-25-1339 41201246

[143] Golden E Rashwan R Woodward EA Sgro A Wang E Sorolla A . The oncogene AAMDC links PI3K-AKT-mTOR signaling with metabolic reprograming in estrogen receptor-positive breast cancer. Nat Commun. (2021) 12:1920. doi: 10.1038/s41467-021-22101-7 33772001 PMC7998036

[144] Jung JH Yang Y Kim Y . Hypoxia-induced SREBP1-mediated lipogenesis and autophagy promote cell survival via fatty acid oxidation in breast cancer cells. Oncol Lett. (2025) 29:175. doi: 10.3892/ol.2025.14921 39975955 PMC11837466

[145] Yin D Fang N Zhu Y Bao X Yang J Zhang Q . Adipocytes-induced ANGPTL4/KLF4 axis drives glycolysis and metastasis in triple-negative breast cancer. J Exp Clin Cancer Res. (2025) 44:192. doi: 10.1186/s13046-025-03458-9 40616161 PMC12231887

[146] Premaratne A Ho C Basu S Khan AF Bawa-Khalfe T Lin CY . Liver X receptor inverse agonist GAC0001E5 impedes glutaminolysis and disrupts redox homeostasis in breast cancer cells. Biomolecules. (2023) 13:345. doi: 10.3390/biom13020345 36830714 PMC9953168

[147] Clusan L Le Goff P Flouriot G Pakdel F . A closer look at estrogen receptor mutations in breast cancer and their implications for estrogen and antiestrogen responses. Int J Mol Sci. (2021) 22:756. doi: 10.3390/ijms22020756 33451133 PMC7828590

[148] Barua D Abbasi B Gupta A Gupta S . XBP1 increases transactivation of somatic mutants of ESR1 and loss of XBP1 reverses endocrine resistance conferred by gain-of-function Y537S ESR1 mutation. Heliyon. (2020) 6(10). doi: 10.1016/j.heliyon.2020.e05217 33088967 PMC7557884

[149] Tecalco-Cruz AC Macias-Silva M Ramirez-Jarquin JO Ramirez-Jarquin UN . Decoding the therapeutic implications of the ERα stability and subcellular distribution in breast cancer. Front Endocrinol. (2022) 13:867448. doi: 10.3389/fendo.2022.867448 35498431 PMC9044904

[150] Gemma C Lai CF Singh AK Belfiore A Portman N Milioli HZ . Induction of the TEAD coactivator VGLL1 by estrogen receptor–targeted therapy drives resistance in breast cancer. Cancer Res. (2024) 84:4283–97. doi: 10.1158/0008-5472.CAN-24-0013 39356622 PMC7616691

[151] Mukhlis Y Amalraj R Natesh J Meeran SM . Noncoding RNAs in metabolic reprogramming of breast cancer: Dietary opportunities and translational implications. In: Non-Coding Rnas: Insights on the Regulatory Genome. Springer Nature Switzerland, Cham (2026). p. 253–89. doi: 10.1007/978-3-032-06948-1_10 41479118

[152] Horie K Takagi K Takeiwa T Mitobe Y Kawabata H Suzuki T . Estrogen-inducible LncRNA BNAT1 functions as a modulator for estrogen receptor signaling in endocrine-resistant breast cancer cells. Cells. (2022) 11:3610. doi: 10.3390/cells11223610 36429038 PMC9688125

[153] Loo SY Syn NL Koh APF Teng JCF Deivasigamani A Tan TZ . Epigenetic derepression converts PPARγ into a druggable target in triple-negative and endocrine-resistant breast cancers. Cell Death Discov. (2021) 7:265. doi: 10.1038/s41420-021-00635-5 34580286 PMC8476547

[154] Xu L Jiang W Li W Guo C Luo L Gao Y . Comparison of a histone deacetylase inhibitor plus exemestane with exemestane alone in hormone receptor-positive advanced breast cancer that progressed on prior endocrine therapy: A meta-analysis. Exp Ther Med. (2022) 24:1–8. doi: 10.3892/etm.2022.11512 35949321 PMC9353490

[155] Achinger-Kawecka J Stirzaker C Portman N Campbell E Chia KM Du Q . The potential of epigenetic therapy to target the 3D epigenome in endocrine-resistant breast cancer. Nat Struct Mol Biol. (2024) 31:498–512. doi: 10.1038/s41594-023-01181-7 38182927 PMC10948365

[156] Maldonado E Morales-Pison S Urbina F Jara L Solari A . Role of the mediator complex and microRNAs in breast cancer etiology. Genes. (2022) 13:234. doi: 10.3390/genes13020234 35205279 PMC8871970

[157] Ogata R Luo Y Fujiwara-Tani R Sasaki R Ikemoto A Maehana K . Long-chain fatty acids alter estrogen receptor expression in breast cancer cells. Int J Mol Sci. (2025) 26:6722. doi: 10.3390/ijms26146722 40724972 PMC12294802

[158] Yan Y Zhang J . Mechanisms of tamoxifen resistance: Insight from long non-coding RNAs. Front Oncol. (2024) 14:1458588. doi: 10.3389/fonc.2024.1458588 39439957 PMC11493607

[159] House RRJ Tovar EA Redlon LN Essenburg CJ Dischinger PS Ellis AE . NF1 deficiency drives metabolic reprogramming in ER+ breast cancer. Mol Metab. (2024) 80:101876. doi: 10.1016/j.molmet.2024.101876 38216123 PMC10844973

[160] Takeiwa T Ikeda K Suzuki T Sato W Iino K Mitobe Y . PSPC1 is a potential prognostic marker for hormone-dependent breast cancer patients and modulates RNA processing of ESR1 and SCFD2. Sci Rep. (2022) 12:9495. doi: 10.1038/s41598-022-13601-7 35681031 PMC9184599

[161] Rosano D Sofyali E Dhiman H Ghirardi C Ivanoiu D Heide T . Long-term multimodal recording reveals epigenetic adaptation routes in dormant breast cancer cells. Cancer Discov. (2024) 14:866–89. doi: 10.1158/2159-8290.CD-23-1161 38527495 PMC11061610

[162] NuzzoPV RodriguesS RibeiroCF TeixeiraIF Nicolo’FanelliG BleveS . Targeting cancer metabolism: Therapeutic potential of the fatty acid synthase (FASN) inhibitors. Crit Rev Oncology/Hematology. (2025), 104910. doi: 10.1016/j.critrevonc.2025.10491040846043

[163] Ismail A Doghish AS Elsadek BE Salama SA Mariee AD . Hydroxycitric acid potentiates the cytotoxic effect of tamoxifen in MCF-7 breast cancer cells through inhibition of ATP citrate lyase. Steroids. (2020) 160:108656. doi: 10.1016/j.steroids.2020.108656 32439410

[164] Huang X Liu B Shen S . Lipid metabolism in breast cancer: From basic research to clinical application. Cancers. (2025) 17:650. doi: 10.3390/cancers17040650 40002245 PMC11852908

[165] Amir M Bakht D Bokhari SFH Yousaf R Iqbal A Nazir H . Lipid metabolism-related genes in gastric cancer: Exploring oncogenic pathways. World J Gastrointestinal Oncol. (2025) 17:106842. doi: 10.4251/wjgo.v17.i8.106842 40837774 PMC12362529

[166] Zhang L Zhu LJ Zhao Y Lei XY Wu DH He KY . STING inhibits the progression of esophageal squamous cell carcinoma by suppressing CPT1A-mediated fatty acid β-oxidation. Acta Pharmacol Sin. (2025) 46:2793–807. doi: 10.1038/s41401-025-01581-z 40394235 PMC12460816

[167] Wang G Chen L Qin S Zhang T Yao J Yi Y . Mechanistic target of rapamycin complex 1: From a nutrient sensor to a key regulator of metabolism and health. Adv Nutr. (2022) 13:1882–900. doi: 10.1093/advances/nmac055 35561748 PMC9526850

[168] Yang Y Zhao P Chen H Tu Y Zhou Y Liu X . Novel strategies against hepatocellular carcinoma through lipid metabolism. Oncol Res. (2025) 33:3247. doi: 10.32604/or.2025.066440 41179299 PMC12573190

[169] Patil P Chaudhary A Bhandare VV Patil VS Beerwala FA Karoshi V . Sesamin regulates breast cancer through reprogramming of lipid metabolism. J Biomol Struct Dyn. (2026) 44:549–69. doi: 10.1080/07391102.2024.2333991 40233124

[170] Zhang Y Liang J Li Z Zuo Y Dai L . ANGPTL4-the link binding lipid metabolism and inflammation. Curr Med Chem. (2025) 32:2931–49. doi: 10.2174/0109298673320024240829070906 39252623

[171] Kim U Chakrabarti R . MDSCs in breast cancer: Metastasis, lipid metabolism, and therapeutics. Mol Cancer Res. (2025) 23:959–70. doi: 10.1158/1541-7786.MCR-24-0838 41085554 PMC12742582

[172] Dai CL Qiu ZY Guo X Yan S Wang AQ Zhao J . Dual targeting of lipogenesis and PUFAs homeostasis by compound kushen injection suppresses breast cancer bone metastasis. Phytomedicine. (2025), 157323. doi: 10.1016/j.phymed.2025.157323 41045621

[173] Cheng C Hu J Mannan R He T Bhattacharyya R Magnuson B . Targeting PIKfyve-driven lipid metabolism in pancreatic cancer. Nature. (2025) 642:776–84. doi: 10.1038/s41586-025-08917-z 40269157 PMC12176661

[174] Banerjee M Zaytseva YY Reusch EM Napier DL Hasani S Rychahou P . FASN inhibition enhances the efficacy of chemotherapy in colorectal cancer by inhibiting the DNA damage response. Cancer Res. (2026) 86(10):2522–36. doi: 10.1158/0008-5472.CAN-25-1917 41661672 PMC13007557

[175] Chetta P Zadra G . Metabolic reprogramming as an emerging mechanism of resistance to endocrine therapies in prostate cancer. Cancer Drug Resistance. (2021) 4:143. doi: 10.20517/cdr.2020.54 35582011 PMC9019185

[176] Guo D Cai S Deng L Xu W Fu S Lin Y . Ferroptosis in pulmonary disease and lung cancer: Molecular mechanisms, crosstalk regulation, and therapeutic strategies. MedComm. (2025) 6:e70116. doi: 10.1002/mco2.70116 39991627 PMC11847630

[177] Han D Ding B Zheng P Tan J Meng Q Zhang W . Albumin nanoparticles co-loaded with dual drugs for enhanced ferroptosis-based cancer therapy through modulating cholesterol metabolism. ACS Nano. (2026) 4(1):143. doi: 10.1021/acsnano.5c17428 41481031 PMC12825383

[178] Yusuf RZ Saez B Sharda A Van Gastel N Yu VW Baryawno N . Aldehyde dehydrogenase 3a2 protects AML cells from oxidative death and the synthetic lethality of ferroptosis inducers. Blood J Am Soc Hematol. (2020) 136:1303–16. doi: 10.1182/blood.2019001808 32458004 PMC7483435

[179] Li Z Yang L Li W Huang W Ma C Sun B . Metabolic reprogramming in urological tumors: New perspectives from tumor metabolic phenotypes to therapeutic targets. Int J Biol Sci. (2025) 21:6926. doi: 10.7150/ijbs.123647 41281744 PMC12631189

[180] Evans KW Yuca E Scott SS Zhao M Paez Arango N Cruz Pico CX . Oxidative phosphorylation is a metabolic vulnerability in chemotherapy-resistant triple-negative breast cancer. Cancer Res. (2021) 81:5572–81. doi: 10.1158/0008-5472.CAN-20-3242 34518211 PMC8563442

[181] Barnabas GD Lee JS Shami T Harel M Beck L Selitrennik M . Serine biosynthesis is a metabolic vulnerability in IDH2-driven breast cancer progression. Cancer Res. (2021) 81:1443–56. doi: 10.1158/0008-5472.CAN-19-3020 33500247 PMC9216326

